# Parallel profiling of DNA methylation and hydroxymethylation highlights neuropathology-associated epigenetic variation in Alzheimer’s disease

**DOI:** 10.1186/s13148-019-0636-y

**Published:** 2019-03-21

**Authors:** Adam R. Smith, Rebecca G. Smith, Ehsan Pishva, Eilis Hannon, Janou A. Y. Roubroeks, Joe Burrage, Claire Troakes, Safa Al-Sarraj, Carolyn Sloan, Jonathan Mill, Daniel L. van den Hove, Katie Lunnon

**Affiliations:** 1 College of Medicine and Health, University of Exeter Medical School, Exeter University, RILD Building Level 4, Royal Devon and Exeter Hospital, Barrack Rd, Exeter, EX2 5DW UK; 2 Department of Psychiatry and Neuropsychology, School for Mental Health and Neuroscience, Maastricht University Medical Centre, Maastricht, The Netherlands; 3 Institute of Psychiatry, King’s College London, London, UK; 4 Nuffield Department of Clinical Neurosciences, University of Oxford, Oxford, UK; 5 Laboratory of Translational Neuroscience, Division of Molecular Psychiatry, Department of Psychiatry, Psychosomatics and Psychotherapy, University of Würzburg, Würzburg, Germany

**Keywords:** Alzheimer’s disease (AD), Brain, Ankyrin 1 (ANK1), DNA methylation (5mC), DNA hydroxymethylation (5hmC), Entorhinal cortex (EC), Epigenetics, Epigenome-wide association study (EWAS), Illumina Infinium Human Methylation 450K microarray (450K array)

## Abstract

**Background:**

Alzheimer’s disease is a progressive neurodegenerative disorder that is hypothesized to involve epigenetic dysfunction. Previous studies of DNA modifications in Alzheimer’s disease have been unable to distinguish between DNA methylation and DNA hydroxymethylation. DNA hydroxymethylation has been shown to be enriched in the human brain, although its role in Alzheimer’s disease has not yet been fully explored. Here, we utilize oxidative bisulfite conversion, in conjunction with the Illumina Infinium Human Methylation 450K microarray, to identify neuropathology-associated differential DNA methylation and DNA hydroxymethylation in the entorhinal cortex.

**Results:**

We identified one experiment-wide significant differentially methylated position residing in the *WNT5B* gene. Next, we investigated pathology-associated regions consisting of multiple adjacent loci. We identified one significant differentially hydroxymethylated region consisting of four probes spanning 104 bases in the *FBXL16* gene. We also identified two significant differentially methylated regions: one consisting of two probes in a 93 base-pair region in the *ANK1* gene and the other consisting of six probes in a 99-base pair region in the *ARID5B* gene. We also highlighted three regions that show alterations in unmodified cytosine: two probes in a 39-base pair region of *ALLC*, two probes in a 69-base pair region in *JAG2*, and the same six probes in *ARID5B* that were differentially methylated. Finally, we replicated significant *ANK1* disease-associated hypermethylation and hypohydroxymethylation patterns across eight CpG sites in an extended 118-base pair region in an independent cohort using oxidative-bisulfite pyrosequencing.

**Conclusions:**

Our study represents the first epigenome-wide association study of both DNA methylation and hydroxymethylation in Alzheimer’s disease entorhinal cortex. We demonstrate that previous estimates of DNA hypermethylation in *ANK1* in Alzheimer’s disease were underestimates as it is confounded by hypohydroxymethylation.

**Electronic supplementary material:**

The online version of this article (10.1186/s13148-019-0636-y) contains supplementary material, which is available to authorized users.

## Background

Alzheimer’s disease (AD) affects approximately 35 million people worldwide [1]. It is a progressive neurodegenerative disease that leads to neuronal cell loss and results in severe cognitive decline. The characteristic hallmarks of AD include neurofibrillary tangles (NFTs) of hyperphosphorylated tau and amyloid beta (Aβ) plaques [2]. The amyloid precursor protein (APP) is normally processed by α- and γ-secretase (non-amyloidogenic pathway), which forms a soluble, non-toxic Aβ fragment called P3. In AD, APP is cleaved by β- and γ-secretase (amyloidogenic pathway) resulting in the formation of a larger Aβ species, which aggregates to form the characteristic plaques associated with the disease. Although Mendelian inheritance of mutations in the *APP* gene and the *PSEN1* and *PSEN2* genes, which encode subunits of the γ-secretase enzyme, have been demonstrated in early-onset familial AD cases, these only account for ~ 5% of disease incidence [3]. The majority of AD cases are sporadic, occur late in life, and have, as yet, no defined etiology, with common single nucleotide polymorphisms (SNPs) accounting for only a third of disease risk [4].

Epigenetic processes mediate the reversible regulation of gene expression, occurring independently of DNA sequence variation and orchestrate a diverse range of important neurobiological processes in the brain. DNA methylation (5-methylcytosine—5mC) is the best characterized and most stable epigenetic modification [5], and recent epigenome-wide association studies (EWAS) have utilized the Illumina Infinium Human Methylation 450K microarray (450K array) to demonstrate robust and reproducible changes in DNA methylation at a number of loci in AD brain [6, 7], including the *ANK1* gene. Although the focus of these studies has been on changes in DNA methylation in AD, the sodium bisulfite (BS) conversion approaches used cannot distinguish DNA methylation from DNA hydroxymethylation (5-hydroxymethylcytosine—5hmC). 5hmC has been previously identified at high levels in the developing [8] and adult brain [9], particularly in neurons [10], and as such may represent an important epigenetic mark to profile in the context of neurodegenerative diseases. Furthermore, levels of 5hmC potentially mask the true abundance of 5mC at specific loci in the genome, confounding existing EWAS analyses of AD.

Recent studies have demonstrated that oxidative BS (OxBS) treatment enables the detection of 5hmC as thymine; therefore, by running matched BS- and OxBS-treated samples in parallel, it is possible to generate a quantitative measurement for total DNA modifications (BS data), DNA methylation (OxBS data), and, by proxy, DNA hydroxymethylation (BS data − OxBS data) and unmodified cytosine (uC) levels (1-BS data) [11]. We have previously utilized this method in conjunction with Illumina 450K arrays to profile 5mC and 5hmC levels in parallel in post-mortem brain tissue from non-demented individuals to demonstrate brain region-specific differences in DNA modifications [11]. Here, we perform an EWAS of 5mC, 5hmC, and uC using the 450K array and brain tissue from 96 donors representing the spectrum of AD pathology defined by Braak staging, a standardized measure of NFT burden determined at autopsy [12], ranging from no AD pathology (Braak 0) to late-stage AD (Braak VI) (Table 1). We looked for an association with Braak stage, rather than clinical diagnosis of disease, as clinical symptoms of the disease are only seen in the middle (limbic) stages of disease (Braak III–IV) [12], while by using Braak stage, we were able to capture epigenetic changes in the earliest stages of disease. From each donor, we analyzed the entorhinal cortex (EC) as this is thought to be the starting point of AD pathology in the cortex with pathology seen here in Braak stage II [12].

**Table 1:** Sample and demographic information for the three cohorts used

	Discovery cohort (450K array)		Validation cohort 1 (450K array)	Validation cohort 2 (pyrosequencing)	
Treatment	BS	OxBS	BS	BS	OxBS
*n* passed QC	91	85	104	96	92
Gender [M/F]	51/40	48/37	42/62	54/42	52/40
Mean age (± SD)	81.2 (9.5)	81.3 (9.5)	84.9 (8.7)	85.0 (7.2)	84.8 (7.3)
Braak stage					
0	8	7	5	5	5
I	3	3	11	6	6
II	11	10	8	37	35
III	6	6	13	0	0
IV	8	7	5	0	0
V	18	17	18	23	22
VI	37	35	44	25	24
Mean PMI [min] (± SD)	2539.5 (1288.1)	2490.7 (1288.5)	1997.6 (1227.0)	2960.6 (1943.8)	2958.9 (1958.7)

The discovery cohort consisted of 450K array BS and OxBS data generated in 96 individuals from the MRC London Brain Bank for Neurodegenerative Disease, with 91 BS arrays and 85 OxBS arrays passing quality control (QC). Validation cohort 1 consisted of previously published 450K array BS data generated in an independent cohort of 104 individuals also from the MRC London Brain Bank for Neurodegenerative Disease [7]. Validation cohort 2 consisted of pyrosequencing BS and OxBS data we generated in an independent cohort of 96 individuals from the Thomas Willis Oxford Brain Collection, with 96 BS and 92 OxBS samples passing QC. For each cohort, we analyzed the entorhinal cortex. Shown for each dataset are the number of samples (*n*) that passed QC, distribution of sex, mean age, Braak stage spread, and postmortem interval (PMI) (± standard deviation (SD))

## Results and discussion

### Highly reproducible alterations in total DNA modifications are detectable in AD EC

**Fig. 1:**
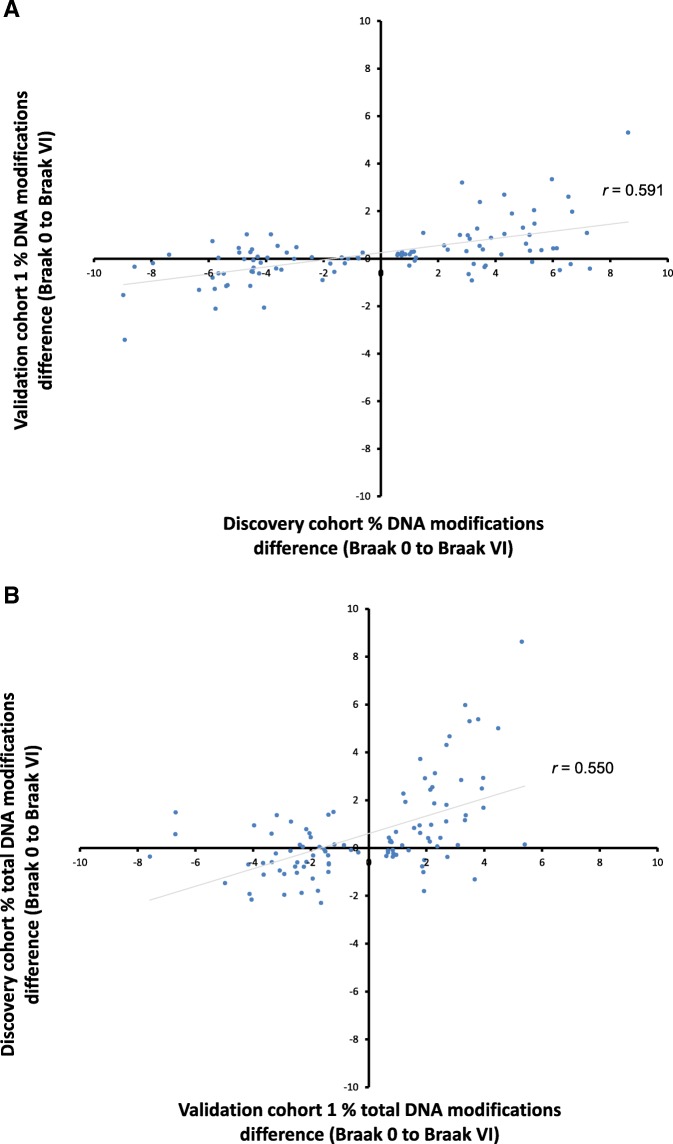
Braak-associated differentially modified positions identified in the EC in BS data in this study are consistent with those identified in previous analyses of AD brain. **a** There was a consistent direction of effect for the top 100 Braak-associated differentially modified positions identified in the EC in BS data in the “discovery cohort” when compared with the same probes in a previously published dataset of Braak-associated differentially modified positions in the EC from BS data (“validation cohort 1”) (sign test *P* = 2.04 × 10^−4^), with the effect sizes highly correlated between studies (*r* = 0.591, *P* = 8.92 × 10^−11^). **b** Similarly, there was a consistent direction of effect for the previously published top 100 Braak-associated differentially modified positions in the EC in BS data (“validation cohort 1”) when compared with the same probes in the current study (sign test *P* = 2.04 × 10^−4^), with the effect sizes highly correlated between studies (*r* = 0.550, *P* = 2.95 × 10^−9^)

Previous studies have used BS DNA in conjunction with the 450K array to identify loci that show AD-associated alterations in total DNA modifications [6, 7]. To assess the reproducibility of our results, we first compared the effect size of the 100 top-ranked Braak-associated differentially modified positions identified in our BS data generated in the “discovery cohort,” with BS data previously published on an independent set of samples from the EC in AD (“validation cohort 1”) [7]. Across these sites, there were significant consistent effects between the two studies (Fig. 1a: sign test *P* = 2.04 × 10^−4^), with the effect sizes highly correlated between studies (*r* = 0.591, *P* = 8.92 × 10^−11^). There were also significant consistent effects across studies for the 100 top-ranked EC Braak-associated differentially modified positions identified in the previous study [7] (Fig. 1b: sign test *P* = 2.04 × 10^−4^), with the effect sizes highly correlated between studies (*r* = 0.550, *P* = 2.95 × 10^−9^). This suggests that although different BS conversion methods and donors were used, there are consistent and reproducible changes in total DNA modifications in the EC of donors with AD.

### Locus-specific changes in DNA methylation and hydroxymethylation occur in AD EC

**Fig. 2:**
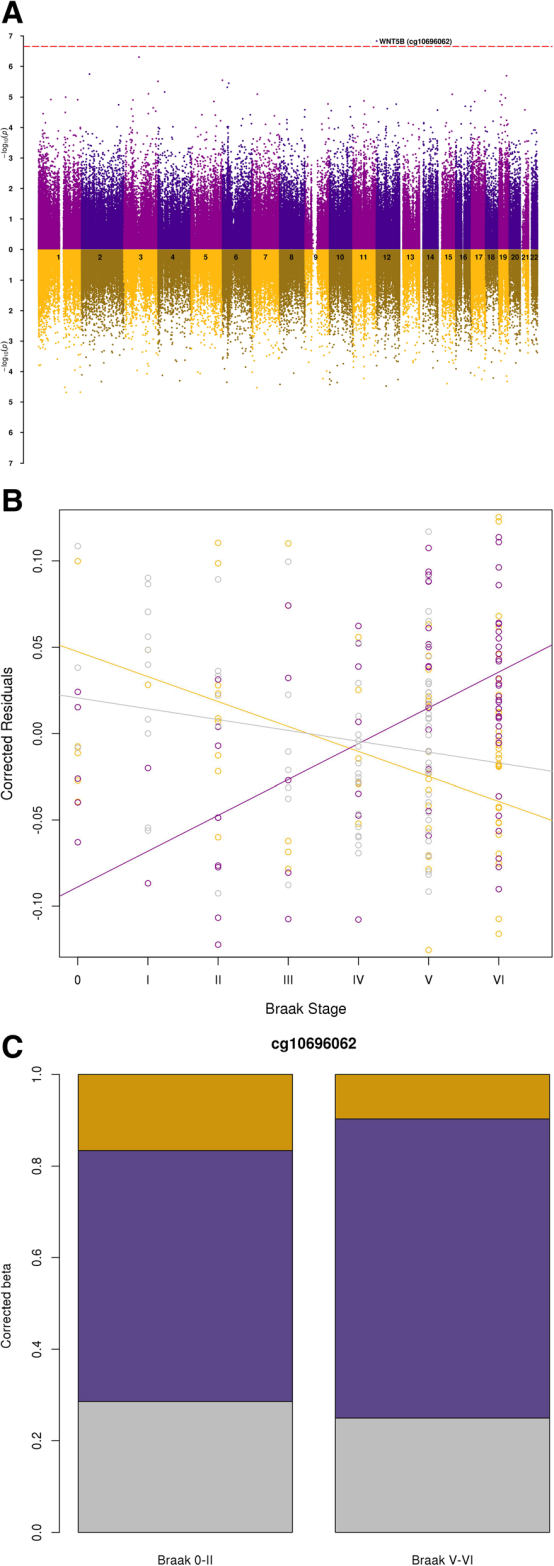
Alterations in DNA methylation at cg10696062 in *WNT5B* are associated with AD neuropathology in the EC. **a** A Manhattan plot of association between DNA methylation (purple, above *X*-axis) and DNA hydroxymethylation (yellow, below *X*-axis) with Braak stage in the EC highlights associations at loci across the genome. One DMP in *WNT5B* (cg10696062) was experiment-wide significant (*P* < 2.4 × 10^− 7^). With increasing Braak stage cg10696062 showed an experiment-wide significant increase in 5mC (*P* = 1.47 × 10^−7^), in parallel with a nominally significant decrease in 5hmC (*P* = 1.56 × 10^−4^) and uC (*P* = 0.02). **b** Scatter plot of residual values, corrected for age, sex, and neuron/glia proportion, against Braak stage for cg10696062 with purple circles representing 5mC levels, yellow circles representing 5hmC levels, and gray circles representing uC levels. Regression lines are shown for 5mC (purple line), 5hmC (yellow line), and uC levels (gray line). **c** Stack chart depicting differences in adjusted beta value (controlling for age, sex, and neuron/glia proportion) between individuals with low (Braak Stage 0–II) and high (Braak Stage V–VI) pathology for 5mC (purple), 5hmC (yellow), and uC (gray) levels

We were interested to identify specific loci that showed Braak-associated alterations in levels of 5mC, 5hmC, or uC in the EC (Fig. 2a). We identified one Braak-associated differentially methylated position (DMP) (cg10696062) that was experiment-wide significant (*P* < 2.4 × 10^−7^), residing within the first intron of the largest isoform of *WNT5B*, a gene which is known to have four isoform variants in humans (Additional file 1: Table S1). We observed a 12.95% change (Δ) in DNA methylation levels between individuals with Braak 0 and Braak VI, with increased 5mC seen with increased disease severity (*P* = 1.47 × 10^− 7^), with a concurrent nominal decrease in 5hmC (*Δ* = − 9.04%, *P* = 1.56 × 10^−4^) and uC (*Δ* = − 3.94%, *P* = 0.02) (Fig. 2b, c). *WNT5B* is part of the *WNT* gene family, and disruption of the *WNT* signaling pathway has been previously implicated in neurodegeneration and AD [13, 14]. Research specifically into a role for *WNT5B* in AD is limited; one study profiled gene expression for all *WNT* genes in EC and showed no difference in *WNT5B* between AD cases and non-demented controls [15]. However, the study was limited to only 10 individuals per group, which highlights the need for further research into this gene. We observed no experiment-wide significant Braak-associated differentially hydroxymethylated positions (DHPs) (Additional file 1: Table S2) or differentially unmodified cytosine positions (DUPs) (Additional file 1: Table S3) in the EC. It is possible that we did not identify any experiment-wide significant DHPs for two reasons. First, we have previously shown that 5hmC is significantly depleted in the proximal promoter of genes [11], and 40% of probes on the 450K array primarily map to these locations [16]. Second, we used a stringent cutoff for measuring 5hmC to avoid false positive results, only including samples in the 5hmC analysis if they had a mean beta value > 0.1 on both the BS and oxBS 450K arrays and if 5hmC was present in more than half of the sample population, meaning that only 219,435 probes were used for the 5hmC analysis (see the “Methods” section).

### Regional analyses identify adjacent DMPs, DHPs, and DUPs

**Fig. 3:**
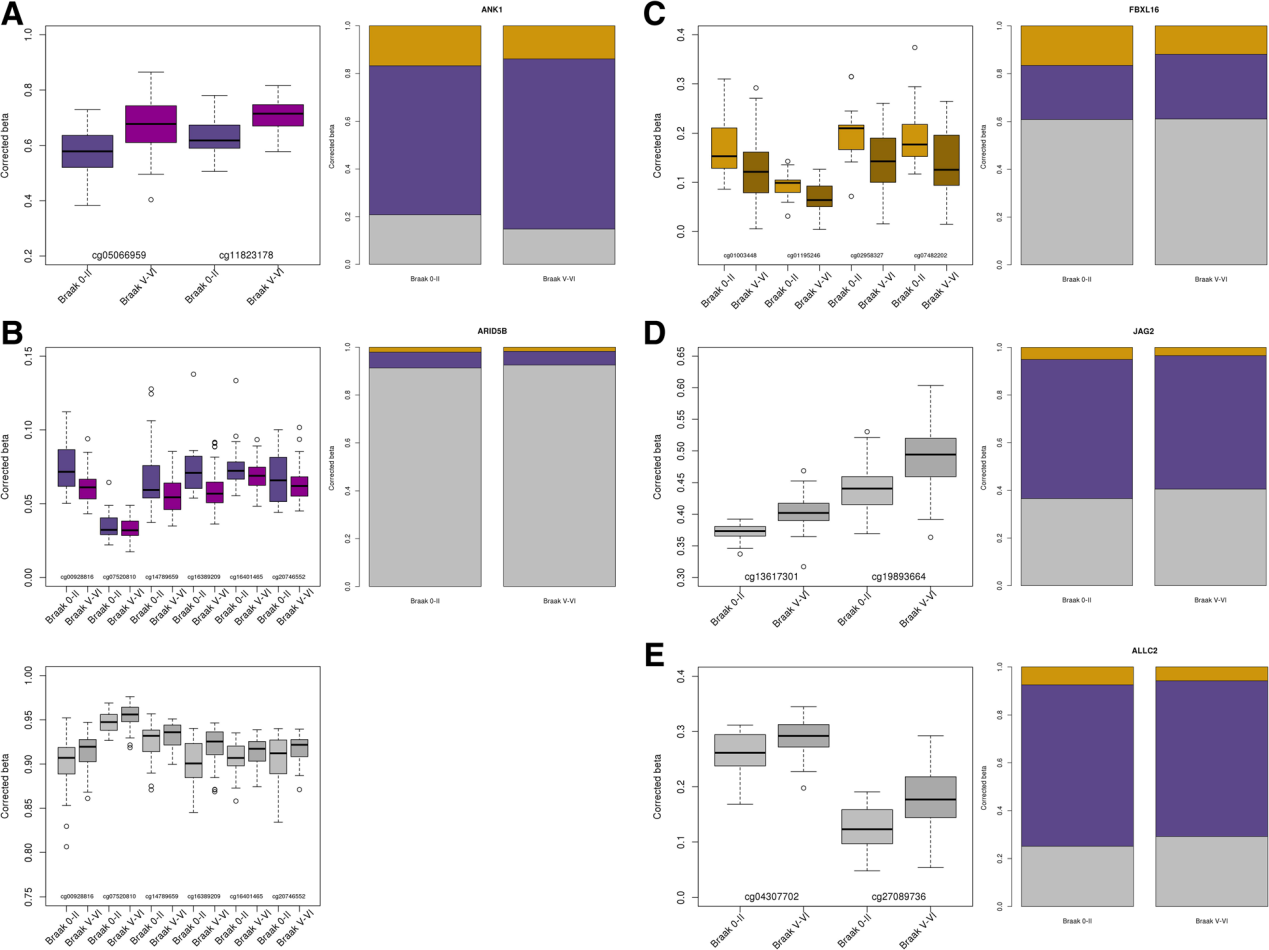
Regional alterations in 5mC, 5hmC, and uC across adjacent sites are associated with AD neuropathology in the EC. **a** A differentially methylated region (DMR) was seen spanning two sites (cg05066959 and cg11823178) in the *ANK1* gene. **b** Within the *ARID5B* gene, a DMR and a differentially unmodified cytosine region (DUR) were identified spanning the same six sites (cg00928816, cg07520810, cg14789659, cg16389209, cg16401465, and cg20746552). **c** One differentially hydroxymethylated region (DHR) was identified, residing within the *FBXL16* gene and containing four CpG sites (cg01003448, cg01195246, cg02958327, cg07482202). An additional two DURs were also identified: **d** one residing within the *JAG2* gene, which covered two loci (cg13617301 and cg19893664) and **e** another residing within the *ALLC* gene, also spanning two loci (cg04307702 and cg27089736). **a**–**e** Left panel shows box plot of adjusted beta value for individual loci making up the DMR, DHR, or DUR, adjusting for the co-variates of age, sex, and neuron/glia proportion, for individuals with low Braak stage (0–II) and high Braak stage (V–VI). Probes in a DMR are shown in purple, DHR in yellow, and DUR in gray. Right panel shows stack chart of differences in adjusted beta value averaged across all the probes in the region, after adjusting for the co-variates of age, sex, and neuron/glia proportion between individuals with low Braak stage (0–II) and high Braak stage (V–VI). 5mC levels are shown in purple, 5hmC levels are shown in yellow, and uC levels are shown in gray

Next, we performed an analysis to identify spatially correlated regions consisting of > 2 neighboring DMPs (differentially methylated regions—DMRs), DHPs (differentially hydroxymethylated regions—DHRs), or DUPs (differentially unmodified regions—DURs) that had a Sidak-corrected *P* value < 0.05 within a 500-bp sliding window (*comb-p* [17]). We identified two DMRs (Table 2 (A)): a two-probe DMR in the *ANK1* gene (Fig. 3a) (Sidak-corrected *P* = 6.50 × 10^−4^) and a six-probe DMR spanning 99 bp in the *ARID5B* gene (Fig. 3b) (Sidak-corrected *P* = 1.18 × 10^−3^). The two DMPs within the *ANK1* DMR have previously been nominated as DMPs in AD cortex [6, 7]. *ARID5B* has not been nominated in previous EWAS and encodes a DNA binding protein, which when associated with phosphorylated PHF2 becomes demethylated, and allows the PHF2-ARID5B complex to function as a histone H3K9Me2 demethylase [18] and increase the target gene’s expression. Interestingly, genetic variation in *ARID5B* has been shown to be associated with AD in two genome-wide association studies (GWAS) [19, 20]. However, these studies showed opposing effects of the SNP (rs2588969) on AD risk. A study has since genotyped rs2588969 and another SNP from the Hollingworth et al. GWAS study (rs4948288) and demonstrated a significant association with AD [21]. However, these effects were lost when the data was adjusted for the co-variates of age at diagnosis, sex, and *APOE* ε4 status, which the authors believed could account for the discrepancy between the two GWAS reports. We found one DHR (Table 2 (B)) consisting of four probes in a 104-bp region of the *FBXL16* gene (Fig. 3c) (Sidak-corrected *P* = 1.55 × 10^−4^). *FBXL16* is a member of the F-box protein family, and expression of this gene has been shown to be upregulated fourfold in a rat model of Parkinson’s disease (PD) [22]. However, a microarray study of acutely isolated microglia from the cortex of an AD mouse model showed a 2.6-fold downregulation in the AD transgenic animals compared to aged wild-type control mice [23]. We identified three DURs (Table 2 (C)): a two-probe DUR in *JAG2* (Fig. 3d) (Sidak-corrected *P* = 1.75 × 10^−5^), a two-probe DUR in *ALLC* (Fig. 3e) (Sidak-corrected *P* = 1.61 × 10^−3^), and a six-probe DUR in *ARID5B* (Fig. 3b) (Sidak-corrected *P* = 2.49 × 10^−4^), covering the same 99-bp region where we observed a DMR. JAG2 is a member of the Notch family and can be cleaved by ADAM10, which is the constitutive α-secretase involved in the non-amyloidogenic processing of APP [24]. Interestingly, He et al. also showed that JAG2 is only weakly cleaved by BACE1, which is the β-secretase enzyme responsible for the amyloidogenic processing of APP.

**Table 2:** Identification of multi-probe regions associated with Braak stage

A					
Position	Gene name	Probes in DMR	Putative region *P*	Sidak-corrected *P*	Probes in DMR
8:41519307-41519400	*ANK1*	2	8.51E−08	6.50E−04	cg05066959 cg11823178
10:63809072-63809171	*ARID5B*	6	1.64E−07	1.18E−03	cg00928816 cg07520810 cg14789659 cg16389209 cg16401465 cg20746552
B					
Position	Gene name	Probes in DHR	Putative region *P*	Sidak-corrected *P*	Probes in DHR
16:745662-745766	*FBXL16*	4	3.75E−08	1.55E−04	cg01003448 cg01195246 cg02958327 cg07482202
C					
Position	Gene name	Probes in DUR	Putative region *P*	Sidak-corrected *P*	Probes in DUR
14:105619633-105619702	*JAG2*	2	1.70E−09	1.75E−05	cg13617301 cg19893664
10:63809072-63809171	*ARID5B*	6	3.46E−08	2.49E−04	cg00928816 cg07520810 cg14789659 cg16389209 cg16401465 cg20746552
2:3714277-3714316	*ALLC*	2	8.84E−08	1.61E−03	cg04307702 cg27089736

Using *Comb-p*, we identified spatially correlated *P* values within a 500-bp sliding window [17]. DMRs are shown in (A), DHRs are shown in (B), and DURs are shown in (C). Shown are regions with a Sidak-corrected *P* value < 0.05

### *ANK1* shows differential DNA methylation in AD

**Fig. 4:**
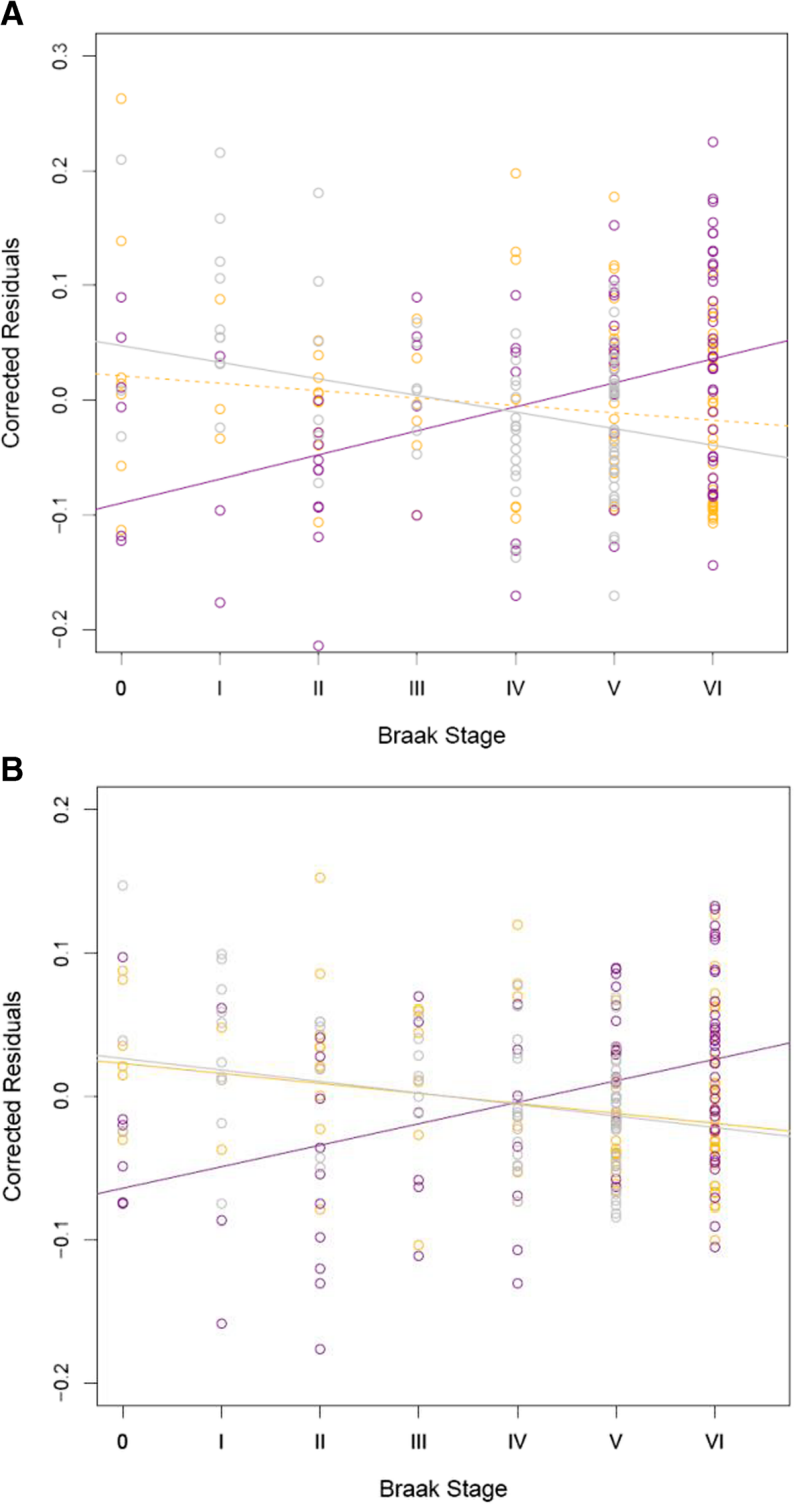
Two probes within *ANK1* are differentially methylated with respect to Braak stage. Shown are beta values for **a** cg05066959 and **b** cg11823178 when adjusted for the co-variates of age, sex, and neuron/glia proportion (*Y*-axis), against Braak stage (*X*-axis). Purple circles represent 5mC levels, yellow circles represent 5hmC levels, and gray circles represent uC levels. Regression lines are shown for 5mC (purple line), 5hmC (yellow line), and uC (gray line) levels. Solid regression lines indicate *P* < 0.05, while dashed lines indicate *P* > 0.05

Interestingly, the two DMPs within the *ANK1* DMR have previously been nominated as DMPs in AD EC in another EWAS [7]. Although these studies claimed to identify neuropathology-associated hypermethylation at both loci, they were actually unable to discriminate between 5mC and 5hmC due to the utilization of BS-treated DNA and, as such, represent a sum of both modifications. In the current study, we demonstrate nominally significant hypermethylation at both loci (Fig. 4a: cg05066959: *∆* = 13.10, *P* = 1.13 × 10^−4^ and Fig. 4b: cg11823178: *∆* = 9.39, *P* = 5.48 × 10^−5^), in parallel with a nominally significant decrease in uC (cg11823178: ∆ = − 5.03, *P* = 1.19 × 10^−3^; cg05066959: *∆ =* − 9.06, *P* = 1.18 × 10^−4^), and a nominally significant decrease in 5hmC at cg11823178 (∆ = − 4.36, *P =* 0.02), with a non-significant decrease in 5hmC at cg05066959 (*∆ =* − 4.04, *P* = 0.14). These figures highlight how alterations in 5mC, 5hmC, and uC are seen early in the disease process. Our findings indicate that previous studies of DNA methylation in *ANK1* have underestimated increments in 5mC in disease due to confounding by 5hmC and could suggest the loss of active DNA demethylation of *ANK1* in AD, as both 5hmC and uC are lower in disease.

### Oxidative-bisulfite pyrosequencing validation of ANK1

**Fig. 5:**
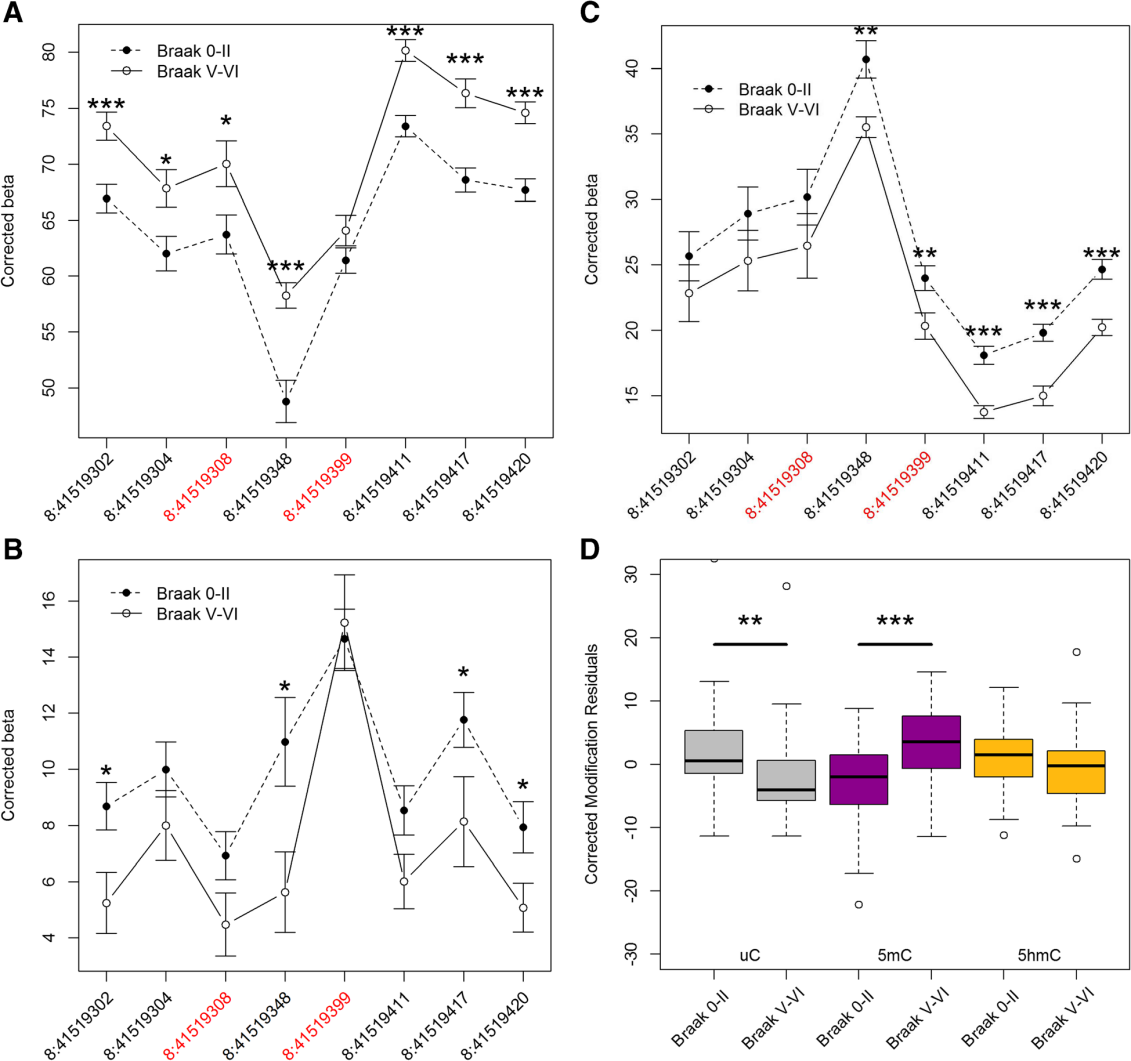
Sites in *ANK1* are characterized by significant DNA hypermethylation and hypohydroxymethylation in AD across an extended region. Using BS and OxBS pyrosequencing, we determined **a** 5mC, **b** 5hmC, and **c** uC levels in the EC in Braak V–VI samples compared to Braak 0-II in “validation cohort 2.” We assayed a 118-bp region containing cg05066959 (chr8:41519308) and cg1182378 (chr8:41519399). We demonstrated significant (*P* < 0.05) neuropathology-associated hypermethylation at seven of the eight CpG sites, significant hypohydroxymethylation at four of the eight CpG sites, and significantly decreased uC at five of the eight sites. **d** Global analysis of all sites within the 118 bp amplicon region highlighted a significant decrease in uC levels (*P* = 6.72 × 10^−3^) and increase in 5mC (*P* = 1.23 × 10^−5^) and a trend towards a decrease in 5hmC (*P* = 0.058) in individuals with Braak V–VI, compared to Braak 0–II. Data is represented as mean (± SEM) Key: *P < 0.05, **P < 0.01, ***P < 0.005

To further investigate whether *ANK1* epigenetic changes associated with neuropathology involve DNA hypermethylation, and not hyperhydroxymethylation, we used OxBS pyrosequencing to quantify DNA modifications across an extended region of 118 bp spanning eight CpG sites, including cg11823178 and cg05066959. DNA used for this study was from an independent collection of EC tissue (*n* = 96) obtained from the Thomas Willis Oxford Brain Collection (“validation cohort 2”) (Table 1) [25]. In this cohort, we did not have access to Braak stage III or IV samples, and as such analyzed data using a case (Braak V–VI)/control (Braak 0–II) analysis model (see the “Methods” section). Of the eight CpG sites assessed, seven were characterized by nominally significant (*P* < 0.05) AD-associated hypermethylation (Fig. 5a). Interestingly, significant AD-associated hypohydroxymethylation was also seen at four of the eight CpG sites (Fig. 5b), although the two loci covered by the 450K array showed no significant difference, further confirming our 450K array findings. We observed a significant decrease in uC at four of the eight sites (Fig. 5c). When we averaged 5mC, 5hmC, and uC levels across the eight CpG sites in the 118-bp region, we saw a significant 6.01% increase in 5mC (*P* = 1.23 × 10^−5^), a significant 3.87% decrease in uC (*P* = 6.72 × 10^−3^), and a trend towards a 2.09% decrease in 5hmC (*P* = 0.058) in individuals with Braak stage V–VI, compared to Braak stage 0–II (Fig. 5d). This data confirms our array-based results and further illustrates that previous studies of *ANK1* DNA modifications in AD have underestimated disease-associated changes in 5mC.

Several recent studies have reported the links between epigenetic alterations at the *ANK1* locus and the development of AD [6, 7, 26]. A recent study showed that alterations in total DNA modifications across the 118-bp amplicon in *ANK1* are seen in the EC in individuals with AD, Huntington’s disease (HD), and PD [27]. Interestingly, individuals with dementia with Lewy bodies (DLB) or vascular dementia (VaD) only showed increased DNA modifications in individuals with co-existing AD pathology. The ANK1 protein is a plasma-bound membrane protein that contains an ankyrin repeat domain, which modulates interactions between cytoskeletal and membrane proteins [28]. One of the main functions of *ANK1* is compartmentalization and maintenance of the plasma membrane, and it is possible that the altered expression of this gene could lead to cell membrane dysfunction in AD [7]. Recent studies suggest that alterations in *ANK1* in AD are glial-derived; a recent EWAS was performed on sorted neuronal and glial nuclei from the temporal cortex and frontal cortex of AD and control donors and demonstrated that Braak-associated alterations in *ANK1* total DNA modifications are of glial origin [29]. Another study has shown that microglia, the brain’s resident macrophages, display a greater than fourfold increase in *ANK1* expression in AD compared to control brain samples [26], suggesting it is possible that *ANK1* DNA methylation levels will have a downstream effect on immune and inflammatory regulation in the brain. Although DNA methylation in the promoter regions of genes is traditionally thought to bring about gene silencing, recent studies show this relationship is context dependent [30]. The DMR we identified in *ANK1* is within the gene body (exon 42 of the longest isoform), and a number of studies have reported a positive correlation between gene body methylation and expression [31].

## Conclusions

Our study represents, to our knowledge, the first systematic study of 5mC and 5hmC in AD, utilizing independent study cohorts and two independent technologies (OxBS-Illumina 450K array and OxBS-pyrosequencing). Two previous studies of DNA hydroxymethylation in AD have been recently published. The first used a selective chemical labeling technique to enrich for 5hmC, and then sequenced the captured libraries from a small cohort of 30 individuals with either AD, mild cognitive impairment (MCI), or no dementia [32]. The study highlighted a number of AD-associated DHPs and demonstrated that gene body DNA hydroxymethylation was positively correlated with cis-acting gene expression. One caveat of that study was that the analysis was unable to discriminate between 5hmC and 5mC because of the low sequencing resolution. The second study, by Ellison and colleagues, utilized reduced representation hydroxymethylation profiling (RRHP) to analyze 5hmC levels in AD hippocampus [33]. Although this study assessed DNA hydroxymethylation at more than two million sites, the study was limited to just three AD cases and two age-matched control subjects. In our study, the overlap between total modifications (BS data) identified across our sample cohorts and those identified by Lunnon et al. [7], despite the use of independent study cohorts, suggests that our study was adequately powered to detect robust AD-associated differences that can be replicated in other studies. Our analyses from multiple independent cohorts provide further evidence for a role for DNA hypermethylation, coupled with hypohydroxymethylation and decreased uC, across a region in *ANK1* in AD-associated neuropathology. It indicates that previous estimates of hypermethylation in *ANK1* were underestimates as it is potentially confounded by this hypohydroxymethylation. As both 5hmC and uC are lower in disease, this suggests the loss of active DNA demethylation in AD. One limitation of our study is that we are unable to determine the functional effects of the disease-associated epigenetic changes we identified and cannot distinguish whether these are causal or secondary to the disease process. Looking to the future, mechanistic experiments using epigenetic editing in induced pluripotent stem cell (iPSC) models are warranted to explore the changes we identified in *WNT5B*, *ANK1*, *ARID5B*, *FBXL16*, *ALLC*, and *JAG2*. These would use, for example, a modified version of the CRISPR-Cas9 technology to alter DNA methylation at these specific loci, and examine their effect on cell function [34].

## Methods

### Subjects and samples

For BS and OxBS Illumina 450K profiling (“discovery cohort”), we used EC brain samples collected from 96 individuals archived in the MRC London Neurodegenerative Disease Brain Bank (https://www.kcl.ac.uk/ioppn/depts/bcn/our-research/neurodegeneration/brain-bank.aspx). These samples were not utilized in previously published AD EWAS publications [7, 35, 36]. Our first “validation cohort” consisted of previously published BS EWAS data generated in 104 EC brain samples [7]. Our second “validation cohort” (for pyrosequencing) consisted of 96 EC brain archived in the Thomas Willis Oxford Brain Collection (https://oxfordbrc.nihr.ac.uk/research-themes-overview/oxford-biorepository/thomas-willis-brain-collection/) [25]. For the “discovery cohort” and “validation cohort 1,” individuals had varying degrees of AD pathology (Braak Stage 0–VI) and this was used as a measure of degree of pathology in the analyses. For “validation cohort 2,” no Braak stage III–IV samples were available so individuals in this cohort were classified as control (Braak 0–II) or cases (Braak V–VI) for analyses. All samples were dissected by trained specialists, snap-frozen, and stored at − 80 °C. Further information about all samples is provided in Table 1. For the “discovery cohort” and “validation cohort 2,” genomic DNA was isolated from ~ 100 mg of each dissected brain region using a standard phenol-chloroform extraction method and tested for degradation and purity prior to analysis.

### Methylomic and hydroxymethylomic profiling

One microgram of DNA from each sample (“discovery cohort” and “validation cohort 2”) was treated with sodium BS and OxBS in parallel using the true-methyl CEGX 96 kit (CEGX, Cambridge, UK) according to the manufacturer’s standard protocol. Briefly, DNA samples were split, with half being oxidized (OxBS) and the remainder (BS) going through a mock oxidization step, before all being BS treated. All “discovery cohort” samples were then processed using the Illumina Infinium Human Methylation 450K Microarray (450 K array) (Illumina Inc., CA, USA) according to the manufacturer’s instructions, with minor amendments and quantified using an Illumina HiScan System (Illumina, CA, USA). “Validation cohort 2” samples were used for pyrosequencing (see the “Targeted replication using bisulfite pyrosequencing” section).

For the 450K array experiments, matched OxBS- and BS-treated DNA from the same sample were run together on the same 450K array. Samples were assigned a unique code for the purpose of the experiment, were randomized in their OxBS and BS pairs with respect to sex and disease status to avoid batch effects, and processed in batches of 12. Illumina Genome Studio software was used to extract the raw signal intensities of each probe (without background correction or normalization).

### Data analysis

All computations and statistical analyses were performed using R 3.3.2 [37] and Bioconductor 3.5 [38]. Signal intensities were imported into R using the methylumi package [39] as a methylumi object. Initial quality control (QC) checks were performed using functions in the methylumi package to assess concordance between reported and genotyped gender. Probes with common (minor allele frequency (MAF) > 5%) SNPs in the CG or single base extension position or probes that are nonspecific or mismapped were flagged and discarded from our results [40]. Data was pre-processed in the R package *wateRmelon* using the *dasen* function as previously described [41]. The pfilter function was used to remove samples where 5% of sites had a detection *P* value > 0.05. Specific sites were also removed if the beadcount was less than three in 5% of samples or if 1% of the samples had a detection *P* > 0.05 at that position. After these QC steps, 367,480 probes were taken forward for analysis. Array data for each treatment was normalized separately. To calculate the proportion of DNA modifications, we used the maximum likelihood methylation levels (MLML) method described by Qu et al. [42]. We used the MLML function within the MLML2R package [43], which uses combined signals from BS and OxBS arrays as an input and returns the estimated proportion of uC, 5mc, and 5hmc for each CpG site. In previous studies, we observed that the naïve subtraction of the OxBS signal from the BS signal results in a negative value for 5hmC for a proportion of CpG sites, likely resulting from technical variance inherent in the Illumina array protocol [11]. When we assessed the distribution of BS-oxBS values, we revealed an accumulation of negative values mainly when the BS 450K array beta value at that given site is < 0.1. Therefore, only probes with a mean beta value > 0.1 on both the BS and oxBS 450K arrays were included in the MLML method (*N* = 355,360 probes). Finally, for 5hmC, we only used sites in our analysis when 5hmC was present in more than half of the sample population (*N* = 219,435 probes).

For the identification of DMPs, DHPs, and DUPs specifically altered with respect to neuropathological measures of AD, we performed a quantitative analysis where samples were analyzed with respect to Braak stage using a linear regression model, and age, sex and neuron/glia proportions were used as co-variates within the analysis. Neuron/glia proportions were calculated using the *CETS* package in R [44]. We performed four linear regression models looking for an association with Braak stage, while controlling for the three co-variates. The first model looked for an association with total DNA modifications (BS data), which was just used to demonstrate consistent effect sizes compared to another previously published EWAS in EC (Fig. 1), and for this analysis, we used the 91 BS samples that passed QC (Table 1). The other models looked for an association with 5mC (OxBS data), 5hmC (BS − OxBS data), or uC (1-BS data) using the output from the MLML method, and in these analyses, we used the 85 samples where the BS and OxBS data both passed QC (Table 1). For each model, Q-Q plots were assessed to check for *P* value inflation (see Additional file 2: Figure S1). Probes were ranked by *P* value, and loci were deemed to be significant if the *P* value was smaller than the multiple testing threshold for 450K array data (“experiment-wide significance”), which was recently reported to be 2.4 × 10^−7^ [45]. The symbol “Δ” in this manuscript refers to the percentage methylation difference between Braak 0 and Braak VI individuals.

### Differentially methylated regions

To identify DMRs, DHRs, and DURs, we identified spatially correlated *P* values within our data using the Python module *comb-p* [17]. This method works by grouping spatially correlated CpGs within a 500-bp sliding window with a significance threshold of *P* < 0.01 into putative regions with a corresponding (uncorrected) regional *P* value. For each putative region, the module then assigns a Sidak-corrected *P* value to account for multiple testing. DMRs, DHRs, and DURs were considered significant if the Sidak-corrected *P* value was < 0.05.

### Targeted replication using bisulfite pyrosequencing

BS and OxBS pyrosequencing was used to quantify 5mC, 5hmC, and uC across eight individual *ANK1* CpG sites, including cg05066959 and cg11823178, spanning from 41519302 to 41519420 within chromosome 8 (hg19). A single amplicon (246 bp) was amplified using primers designed using the PyroMark Assay Design software 2.0 (Qiagen, UK) as previously described [7], and sequenced using two sequencing primers to maximize coverage across eight CpG sites within a 118-bp region. DNA methylation was quantified in “validation cohort 2” using the PyroMark Q24 system (Qiagen, UK) following the manufacturer’s standard instructions and the Pyro Q24 CpG 2.0.6 software. 5hmC values were calculated by the subtraction of the oxBS signal from the BS signal, uC values were calculated by subtracting the BS signals from 100, and 5mC values were equal to the OxBS signal. Data was adjusted for the effects of age and sex. For this sample cohort, we had no samples with middle stage AD (Braak III–IV). In this instance, rather than performing a linear regression analysis across Braak stages, we instead compared control samples (Braak scores 0–II) to AD samples (Braak scores V–VI) for 5mC, 5hmC, and uC levels at eight individual CpG sites and averaged across the 118-bp amplicon.

## Additional files

Additional file 1: **Tables S1, S2 & S3.** The 100 most significant neuropathology-associated differentially methylated (DMPs), hydroxymethylated (DHPs) and unmodified positions (DUPs) in the EC. Shown for each probe are chromosomal location (hg19), UCSC annotation, and GREAT annotation, with corrected effect size (difference between Braak 0 and Braak VI (Δ)) and corresponding *P* value after adjusting for the co-variates of age, sex, and neuron/glia proportions. Probes where the 5hmC was below the level of detection are represented by “-”. All *P* values < 0.05 are shown in bold. (PDF 1344 kb)

Additional file 2: **Figure S1.** Quantile-quantile (Q-Q) plots of expected versus observed *P* value to check for inflation in linear regression analyses. Q-Q plots are shown for (**A**) unmodified cytosine (uC) (1-BS data), (**B**) 5mC (OxBS data), (**C**) 5hmC (BS – OxBS data), and (**D**) total DNA modifications (BS data). (PDF 125 kb)

## Abbreviations

450K array: Illumina 450K methylation array
5hmC: 5-Hydroxymethylctosine
5mC: 5-Methylcytosine
AD: Alzheimer’s disease
Aβ: Amyloid beta
BS: Bisulfite
CGI: CpG island
DHP: Differentially hydroxymethylated position
DHR: Differentially hydroxymethylated region
DMP: Differentially methylated position
DMR: Differentially methylated region
DUP: Differentially unmodified cytosine position
DUR: Differentially unmodified cytosine region
EC: Entorhinal cortex
EWAS: Epigenome-wide association study
GWAS: Genome-wide association study
MAF: Minor allele frequency
NFT: Neurofibrillary tangle
OxBS: Oxidative bisulfite
QC: Quality control
SNP: Single nucleotide polymorphism
uC: Unmodified cytosine
