# Supplementary material for: Parallel profiling of DNA methylation and hydroxymethylation highlights neuropathology-associated epigenetic variation in Alzheimer’s disease
Source: Clin Epigenetics. 2019 Mar 21;11:52. doi: 10.1186/s13148-019-0636-y (PMC6429761; doi:10.1186/s13148-019-0636-y)
Supplement: Supplementary file 1 — Tables S1, S2 & S3. The 100 most significant neuropathology-associated differentially methylated (DMPs), hydroxymethylated (DHPs) and unmodified positions (DUPs) in the EC. Shown for each probe are chromosomal location (hg19), UCSC annotation, and GREAT annotation, with corrected effect size (difference between Braak 0 and Braak VI (Δ)) and corresponding P value after adjusting for the co-variates of age, sex, and neuron/glia proportions. Probes where the 5hmC was below the level of detection are represented by “-”. All P values < 0.05 are shown in bold. (PDF 1344 kb) [file 13148_2019_636_MOESM1_ESM.pdf]

Table S1 The 100 most significant neuropathology-associated differentially methylated positions (DMPs) in the EC.

Shown for each probe are chromosomal location (hg19), UCSC annotation, GREAT annotation, with corrected effect size (difference between Braak 0 and Braak VI (Δ) in 5mC levels) and corresponding P value after adjusting for the co-variates of age, sex, and neuron/glia proportions. Also shown for all probes are Δ and corresponding P value for unmodified cytosine (uC) and 5hmC levels. Probes where the 5hmC was below the level of detection are represented by "-". All P values <0.05 are shown in bold.

Probe information					5mC		uC		5hmC	
Rank	Probe	Position	UCSC Gene Annotation	GREAT Annotation	*P*	Δ	*P*	Δ	*P*	Δ
1	cg10696062	12:1726028	*WNT5B*	*WNT5B*	**1.47E-07**	12.97	**0.0199**	-3.94	**1.56E-04**	-9.04
2	cg15147060	3:88108213	*CGGBP1*	*CGGBP1*	**4.96E-07**	-0.77	**1.31E-03**	0.54	-	-
3	cg18110359	2:45179970		*SIX3*	**1.79E-06**	-1.17	0.5472	0.24	-	-
4	cg02456292	19:42817262	*TMEM145*	*TMEM145*	**2.03E-06**	-1.58	0.0778	0.71	-	-
5	cg17304222	5:180076905	*FLT4*	*FLT4*	**2.85E-06**	-0.98	**3.44E-06**	1.12	-	-
6	cg26332552	3:197402549	*MIR922;KIAA0226*	*KIAA0226*	**3.11E-06**	10.83	**8.26E-04**	-6.02	**0.0228**	-4.81
7	cg02412050	6:35995429	*MAPK14*	*MAPK14*	**3.57E-06**	-0.65	**9.56E-04**	0.61	-	-
8	cg05645927	6:27344393	*ZNF204P*	*ZNF391*	**4.85E-06**	8.05	0.7755	-0.48	**2.83E-04**	-7.57
9	cg17917959	17:79197229	*AZI1*	*AZI1*	**6.14E-06**	1.24	0.1199	-0.36	-	-
10	cg09560294	4:37892962	*TBC1D1*	*TBC1D1*	**6.78E-06**	-0.99	0.0531	0.49	-	-
11	cg19893077	3:142314877	*PLS1*	*ATR*	**7.77E-06**	-1.52	**4.26E-03**	1.04	-	-
12	cg13323756	7:29847108		*WIPF3*	**7.98E-06**	3.98	0.0835	-2.03	0.0508	-1.96
13	cg11706226	15:81391334		*MESDC2*	**8.06E-06**	8.38	0.3847	-1.50	**9.26E-04**	-6.88
14	cg14294321	19:12895288		*JUNB*	**8.33E-06**	5.67	0.3310	-1.46	**0.0228**	-4.20
15	cg13170934	1:156722147	*HDGF*	*HDGF*	**9.99E-06**	-1.22	**6.38E-03**	0.71	-	-
16	cg21808240	19:36822564		*ZFP14*	**1.07E-05**	-0.74	**0.0422**	3.81E-01	-	-
17	cg24516830	1:71694460		*ZRANB2*	**1.20E-05**	8.83	0.2015	-2.13	**2.80E-03**	-6.70
18	cg15624314	1:226251003	*H3F3A;LOC440926*	*H3F3A*	**1.22E-05**	-0.93	**0.0343**	0.57	-	-
19	cg00106685	3:52720133	*GNL3;PBRM1*	*GNL3*	**1.24E-05**	-0.62	**0.0152**	0.40	-	-
20	cg16898576	11:63449499	*RTN3*	*RTN3*	**1.32E-05**	-0.60	**0.0217**	0.37	-	-
21	cg24698371	19:6110817	*RFX2*	*RFX2*	**1.36E-05**	0.97	**1.68E-04**	-0.88	-	-
22	cg05052335	5:130970657	*RAPGEF6*	*RAPGEF6*	**1.39E-05**	-0.67	**1.55E-03**	0.49	-	-
23	cg09103877	17:17713067	*RAI1*	*SREBF1*	**1.40E-05**	15.30	0.9469	-0.11	**7.40E-05**	-15.19
24	cg01888395	9:132145105		*NTMT1*	**1.67E-05**	4.91	0.2887	-1.74	0.0566	-3.17
25	cg14911708	12:114404314	*RBM19*	*RBM19*	**1.70E-05**	0.68	**1.08E-04**	-0.63	-	-
26	cg15235057	19:16683989	*SLC35E1*	*SLC35E1*	**1.79E-05**	-0.82	**0.0288**	0.46	-	-
27	cg00639289	2:213403734	*ERBB4*	*ERBB4*	**1.79E-05**	-0.56	**3.80E-04**	0.49	-	-

Probe information					5mC		uC		5hmC	
Rank	Probe	Position	UCSC Gene Annotation	GREAT Annotation	*P*	Δ	*P*	Δ	*P*	Δ
28	cg27228168	16:54320674	*IRX3*	*IRX3*	**1.99E-05**	-0.73	**1.39E-03**	0.57	-	-
29	cg05341384	16:86331661		*FOXF1*	**2.04E-05**	-3.44	0.2194	0.90	**1.33E-03**	2.54
30	cg03503087	10:118032626	*GFRA1*	*GFRA1*	**2.04E-05**	-0.84	0.1532	0.29	-	-
31	cg11507178	11:118781515	*BCL9L*	*BCL9L*	**2.16E-05**	-1.28	0.3318	0.53	-	-
32	cg21196581	3:169780131	*GPR160*	*GPR160*	**2.23E-05**	7.45	0.4289	-1.15	**1.15E-03**	-6.30
33	cg12366118	12:62860687	*MON2*	*MON2*	**2.40E-05**	-0.79	**4.25E-03**	0.61	-	-
34	cg23044270	3:43659607	*ANO10*	*ANO10*	**2.44E-05**	7.05	0.6136	-0.55	**1.15E-03**	-6.50
35	cg03792042	8:145045235	*PLEC1*	*PARP10*	**2.59E-05**	7.21	0.3255	-2.28	**0.0231**	-4.93
36	cg26412722	21:27012615	*JAM2*	*JAM2*	**2.61E-05**	-1.38	**2.49E-04**	1.30	-	-
37	cg04418576	14:103851554	*MARK3*	*MARK3*	**2.67E-05**	-0.71	**7.43E-03**	0.52	-	-
38	ch.16.83989841F	16:85432340		*GSE1*	**2.75E-05**	4.62	0.2403	-1.17	**0.0179**	-3.46
39	cg22782712	10:9920178			**2.78E-05**	5.99	0.6201	0.58	**4.93E-05**	-6.56
40	cg22902534	3:183892754	*AP2M1*	*AP2M1*	**2.89E-05**	-1.40	**0.0261**	0.68	-	-
41	cg09311052	1:154531418	*UBE2Q1*	*UBE2Q1*	**3.17E-05**	-1.07	**5.25E-03**	0.81	-	-
42	cg25967872	3:141205623	*RASA2*	*RASA2*	**3.47E-05**	1.14	**3.16E-04**	-1.06	-	-
43	cg14164839	1:26823481		*HMGN2*	**3.49E-05**	-5.58	0.2081	1.69	**6.94E-03**	3.89
44	cg15826130	11:67159072	*LOC100130987;RAD9A*	*RAD9A*	**3.63E-05**	-0.82	**0.0150**	0.54	-	-
45	cg04883450	5:61699957	*DIMT1L*	*DIMT1*	**3.78E-05**	-0.66	**0.0119**	0.45	-	-
46	cg20707008	6:160210612	*MRPL18;TCP1*	*TCP1*	**3.79E-05**	-0.86	**0.0364**	0.48	-	-
47	cg05652757	5:139227606	*NRG2*	*PSD2*	**3.90E-05**	-1.10	0.2198	0.46	-	-
48	cg06898199	7:128502890	*ATP6V1F*	*ATP6V1F*	**4.10E-05**	0.97	**1.17E-05**	-1.03	-	-
49	cg14467668	3:52571040	*LOC440957*	*SMIM4*	**4.21E-05**	-0.57	0.0834	0.26	-	-
50	cg04002944	8:143558609	*BAI1*	*BAI1*	**4.44E-05**	-7.51	**3.57E-05**	6.06	0.3478	1.46
51	cg17998333	10:121356721	*TIAL1*	*TIAL1*	**4.46E-05**	-0.74	**0.0155**	0.51	-	-
52	cg03157738	19:11308118	*KANK2*	*KANK2*	**4.56E-05**	-0.59	**1.09E-04**	0.73	-	-
53	cg09811464	9:135465645		*BARHL1*	**4.91E-05**	-2.14	0.1132	1.05	0.1014	1.09
54	cg06734816	6:116422381	*NT5DC1*	*NT5DC1*	**5.14E-05**	-0.70	**3.12E-03**	0.53	-	-
55	cg11823178	8:41519399	*ANK1;MIR486*	*NKX6-3*	**5.48E-05**	9.39	**1.19E-03**	-5.03	**0.0157**	-4.36
56	cg02977761	19:42901279		*CNFN*	**5.78E-05**	-1.12	**6.49E-05**	0.99	-	-
57	cg22809017	5:169373347	*FAM196B;DOCK2*	*FOXI1*	**5.98E-05**	-3.97	0.7351	0.38	**1.34E-03**	3.59
58	cg06121461	18:43684114	*ATP5A1;HAUS1*	*ATP5A1*	**6.15E-05**	1.17	**4.02E-04**	-1.02	-	-
59	cg04332914	3:53381587	*DCP1A*	*DCP1A*	**6.24E-05**	-0.79	**0.0139**	0.57	-	-

Probe information					5mC		uC		5hmC	
Rank	Probe	Position	UCSC Gene Annotation	GREAT Annotation	*P*	Δ	*P*	Δ	*P*	Δ
60	cg21878074	12:13153637	*HTR7P;HEBP1*	*HEBP1*	**6.46E-05**	-1.25	**0.0210**	0.75	-	-
61	cg07170170	12:125672141		*AACS*	**6.93E-05**	-0.75	0.0797	0.40	-	-
62	cg18611777	14:24584409	*DCAF11*	*NRL*	**6.93E-05**	-1.51	**6.93E-04**	1.42	-	-
63	cg15322430	1:40157251	*HPCAL4*	*HPCAL4*	**6.95E-05**	1.71	**1.03E-04**	-1.62	-	-
64	cg06624337	15:47477411		*SEMA6D*	**6.98E-05**	-0.73	**0.0103**	0.54	-	-
65	cg21209091	8:42234975	*DKK4*	*DKK4*	**7.12E-05**	4.55	0.7222	-0.40	**2.04E-03**	-4.15
66	cg16046673	13:51483748	*RNASEH2B*	*RNASEH2B*	**7.13E-05**	-1.17	0.0864	0.63	-	-
67	cg27175943	16:85334190		*KIAA0513*	**7.31E-05**	-11.99	0.1183	4.30	**0.0451**	7.70
68	cg26198430	6:166796458	*BRP44L*	*MPC1*	**7.41E-05**	0.37	**2.68E-03**	-0.30	-	-
69	cg14956593	14:24685421	*MDP1*	*MDP1*	**7.41E-05**	-0.79	0.0933	0.40	-	-
70	cg26666512	16:30934656	*NCRNA00095;FBXL19*	*FBXL19*	**7.54E-05**	1.15	0.0884	-0.65	-	-
71	cg26510723	7:157464230	*PTPRN2*	*DNAJB6*	**7.58E-05**	-7.85	**3.47E-03**	5.30	0.2219	2.56
72	cg24898753	11:61735917	*FTH1*	*FTH1*	**7.58E-05**	-2.00	0.5172	-0.48	-	-
73	cg09015484	9:96929106		*PTPDC1*	**7.60E-05**	-0.86	**7.36E-03**	0.67	-	-
74	cg07919466	19:50833956	*KCNC3*	*KCNC3*	**7.65E-05**	-0.85	0.1763	0.40	-	-
75	cg22439857	7:77166225	*PTPN12*	*PTPN12*	**7.86E-05**	-0.85	**0.0111**	0.65	-	-
76	cg27611665	19:9929320	*FBXL12*	*FBXL12*	**7.95E-05**	-0.46	0.1077	0.20	-	-
77	cg23648516	13:78467552		*EDNRB*	**8.02E-05**	8.79	0.6418	0.70	**1.97E-04**	-9.49
78	cg10624860	11:75945841		*WNT11*	**8.10E-05**	-1.63	0.5663	0.38	-	-
79	cg26463399	19:44949017	*ZNF229*	*ZNF229*	**8.31E-05**	4.80	0.6950	0.30	**5.79E-04**	-5.10
80	cg12776966	19:30021276	*VSTM2B*	*VSTM2B*	**8.53E-05**	-1.07	0.5151	0.31	-	-
81	cg03958979	6:108486387	*NR2E1*	*NR2E1*	**8.58E-05**	-1.99	0.0690	1.05	0.1383	0.94
82	cg09001549	12:129281454	*SLC15A4*	*SLC15A4*	**8.70E-05**	8.59	**2.65E-04**	-5.33	0.1227	-3.26
83	cg07379167	10:131769074		*EBF3*	**8.72E-05**	-1.74	0.0891	0.67	-	-
84	cg22203547	5:145717010		*POU4F3*	**8.94E-05**	-2.29	**2.96E-04**	2.46	0.7733	-0.16
85	cg24007300	15:42264860	*EHD4*	*EHD4*	**8.97E-05**	-0.40	0.1140	0.16	-	-
86	cg04108502	12:56360908	*CDK2;SILV*	*CDK2*	**9.23E-05**	-1.27	0.5042	0.27	-	-
87	cg21580173	6:32940045	*BRD2*	*BRD2*	**9.30E-05**	-0.76	0.2099	0.32	-	-
88	cg22598426	4:85417755	*NKX6-1*	*NKX6-1*	**9.39E-05**	-3.67	0.0640	1.73	**0.0349**	1.93
89	cg05983229	2:204192854	*ABI2*	*ABI2*	**9.42E-05**	0.51	**6.44E-04**	-0.48	-	-
90	cg05813194	6:27342597	*ZNF204P*	*ZNF391*	**9.47E-05**	-1.04	**0.0159**	0.83	-	-
91	cg27460531	17:78791723	*RPTOR*	*CHMP6*	**9.70E-05**	-5.69	0.9386	0.07	**5.30E-04**	5.62

Probe information					5mC		uC		5hmC	
Rank	Probe	Position	UCSC Gene Annotation	GREAT Annotation	*P*	Δ	*P*	Δ	*P*	Δ
92	cg13844922	14:65409659	*GPX2*	*GPX2*	**9.80E-05**	6.08	0.6622	-0.56	**7.75E-03**	-5.52
93	cg22596806	1:241517815	*RGS7*	*RGS7*	**9.90E-05**	-6.92	0.1763	2.30	**0.0283**	4.62
94	cg08261430	5:26237728		*CDH9*	**9.93E-05**	5.30	**1.30E-03**	-1.91	**7.45E-03**	-3.38
95	cg25788088	4:1546465		*FAM53A*	**9.99E-05**	-9.01	0.2855	1.45	**1.64E-03**	7.56
96	cg10861731	10:50395528	*C10orf128*	*C10orf128*	**1.01E-04**	6.95	0.8023	-0.53	**1.78E-03**	-6.42
97	cg11006962	11:59323675		*OR4D9*	**1.01E-04**	-2.29	0.5279	0.49	**4.11E-02**	1.79
98	cg10457504	17:19281708	*MAPK7*	*MAPK7*	**1.01E-04**	-0.91	**0.0294**	0.58	-	-
99	cg17565360	11:4115872	*RRM1*	*RRM1*	**1.02E-04**	-1.19	**0.0213**	0.72	-	-
100	cg17026303	3:38495161	*LOC100128640;ACVR2B*	*ACVR2B*	**1.02E-04**	-0.97	**0.0159**	0.61	-	-

Supplementary Table 2: The 100 most significant neuropathology-associated differentially hydroxymethylated positions (DHPs) in the EC. Shown for each probe are chromosomal location (hg19), UCSC annotation, GREAT annotation, with corrected effect size (difference between Braak 0 and Braak VI (Δ) in 5hmC levels) and corresponding P value after adjusting for the co-variates of age, sex, and neuron/glia proportions. Also shown for all probes are Δ and corresponding P value for unmodified cytosine (uC) and 5mC levels alone. All P values <0.05 are shown in bold.

Probe Information					5hmC		uC		5mC	
Rank	Probe	Position	UCSC Gene Annotation	GREAT Annotation	*P*	Δ	*P*	Δ	*P*	Δ
1	cg20066612	1:160050948	*KCNJ9*	*KCNJ9*	**2.07E-05**	5.19	**0.0003**	-2.92	**0.0465**	-2.27
2	cg08240832	1:241791729	*OPN3*	*CHML*	**2.11E-05**	-9.63	**0.0227**	2.68	**0.0001**	6.95
3	cg15700661	7:26958919		*SKAP2*	**2.96E-05**	-5.04	0.1507	1.40	**0.0027**	3.64
4	cg19370451	1:152386561	*CRNN*	*CRNN*	**3.04E-05**	-5.13	**0.0055**	2.64	**0.0456**	2.49
5	cg11835619	12:56475064	*ERBB3*	*PA2G4*	**3.33E-05**	3.87	0.8220	0.34	**0.0072**	-4.21
6	cg05441518	10:1739453	*ADARB2*	*IDI1*	**3.75E-05**	7.95	**0.0036**	-3.90	**0.0057**	-4.04
7	cg19005236	8:1571105	*DLGAP2*	*CLN8*	**3.78E-05**	-9.71	0.1690	2.63	**0.0020**	7.08
8	cg15481603	15:51977697	*SCG3*	*SCG3*	**4.03E-05**	-9.38	**0.0008**	4.52	**0.0088**	4.85
9	cg06174962	10:44351762		*HNRNPA3P1*	**4.37E-05**	-8.18	**0.0485**	2.08	**0.0006**	6.10
10	cg06657721	6:25027618		*FAM65B*	**4.46E-05**	-7.92	0.5092	0.76	**0.0015**	7.16
11	cg23407561	2:113544232	*IL1A*	*IL1A*	**4.67E-05**	-5.37	**0.0028**	2.93	**0.0154**	2.44
12	cg08182454	19:46529189		*PGLYRP1*	**4.72E-05**	-10.08	**0.0193**	4.13	**0.0027**	5.95
13	cg22782712	10:9920178		*NONE*	**4.93E-05**	-6.57	0.6201	0.58	**0.0000**	5.99
14	cg20711695	4:124698165	*LOC285419*	*SPRY1*	**4.98E-05**	-7.91	**0.0070**	3.15	**0.0133**	4.76
15	cg16337430	12:124876433	*NCOR2*	*NCOR2*	**5.45E-05**	-6.78	**0.0327**	3.61	0.0579	3.16
16	cg07287508	17:59471566		*TBX2*	**5.55E-05**	4.40	0.8440	-0.25	**0.0056**	-4.16
17	cg10936057	10:708425	*DIP2C;C10orf108*	*DIP2C*	**5.64E-05**	8.90	0.1289	-3.29	**0.0191**	-5.62
18	cg14546505	9:102863959	*INVS*	*ERP44*	**5.86E-05**	-6.80	0.0550	2.89	**0.0231**	3.91
19	cg16013966	11:62380545	*EML3;ROM1*	*EML3*	**5.96E-05**	3.59	**0.0000**	-3.47	0.8378	-0.12
20	cg00416882	18:44495671	*PIAS2*	*ST8SIA5*	**6.13E-05**	-8.20	0.0625	2.84	**0.0009**	5.36
21	cg19776453	19:43033801	*CEACAM1*	*CEACAM1*	**7.13E-05**	4.73	0.0817	-2.14	**0.0012**	-2.60
22	cg09103877	17:17713067	*RAI1*	*SREBF1*	**7.40E-05**	-15.19	0.9469	-0.11	**0.0000**	15.30
23	cg10766585	4:24801110	*SOD3*	*SOD3*	**7.52E-05**	-8.24	0.9853	0.03	**0.0008**	8.21
24	cg22166977	2:1159491	*SNTG2*	*TPO*	**7.80E-05**	-7.08	0.2066	1.68	**0.0014**	5.40
25	cg07283011	3:133970158	*RYK*	*RYK*	**8.47E-05**	-9.70	0.1189	2.64	**0.0008**	7.06
26	cg18616279	5:51813651		*PELO*	**8.72E-05**	-4.35	0.4087	0.81	**0.0005**	3.54
27	cg17337106	8:80577176	*STMN2*	*STMN2*	**8.87E-05**	-6.71	**0.0146**	2.81	**0.0025**	3.90

Probe Information					5hmC		uC		5mC	
Rank	Probe	Position	UCSC Gene Annotation	GREAT Annotation	*P*	Δ	*P*	Δ	*P*	Δ
28	cg02610274	16:85435753		*KIAA0182*	**9.09E-05**	-8.57	0.2952	1.32	**0.0006**	7.26
29	cg02345908	10:1814106		*ADARB2*	**9.34E-05**	-5.83	0.1295	1.73	**0.0019**	4.10
30	cg06719973	7:207666	*FAM20C*	*FAM20C*	**9.53E-05**	-9.83	0.1039	2.23	**0.0012**	7.60
31	cg20699036	5:38556796	*LIFR*	*LIFR*	**9.64E-05**	-2.24	0.4359	0.39	**0.0010**	1.85
32	cg14023291	9:124615180	*TTLL11*	*TTLL11*	**1.04E-04**	-10.97	0.0891	2.84	**0.0036**	8.13
33	cg22761670	17:74268201		*RNF157*	**1.07E-04**	-7.19	0.0864	2.64	**0.0107**	4.55
34	cg16933762	3:77147109	*ROBO2*	*ROBO2*	**1.10E-04**	-8.42	0.9271	-0.15	**0.0001**	8.57
35	cg17848546	1:209605304	*LOC642587;MIR205*	*CAMK1G*	**1.12E-04**	-6.97	**0.0116**	3.20	**0.0162**	3.77
36	cg02170784	16:1034527	*SOX8*	*SSTR5*	**1.23E-04**	4.17	0.2897	-1.36	**0.0066**	-2.81
37	cg03276149	6:11651032		*TMEM170B*	**1.23E-04**	-8.15	**0.0430**	3.21	**0.0052**	4.95
38	cg23895846	14:77938718		*AHSA1*	**1.32E-04**	-6.51	0.7518	0.56	**0.0016**	5.95
39	cg02570361	17:42280026		*ATXN7L3*	**1.35E-04**	-6.59	**0.0001**	4.76	0.2384	1.82
40	cg02021157	11:2644078	*KCNQ1*	*KCNQ1*	**1.38E-04**	-6.83	**0.0096**	3.67	**0.0323**	3.16
41	cg24485049	6:132968496	*TAAR1*	*TAAR1*	**1.41E-04**	-6.77	**0.0060**	3.55	**0.0301**	3.22
42	cg15392618	1:240368441	*FMN2*	*FMN2*	**1.42E-04**	-7.39	**0.0089**	4.11	**0.0466**	3.28
43	cg00509451	11:64811775	*SAC3D1*	*SAC3D1*	**1.47E-04**	8.88	**0.0020**	-4.63	**0.0304**	-4.25
44	cg07269003	3:142681516	*PAQR9*	*PAQR9*	**1.53E-04**	-7.24	**0.0335**	2.78	**0.0224**	4.46
45	cg10696062	12:1726028	*WNT5B*	*FBXL14*	**1.56E-04**	-9.04	**0.0199**	-3.94	**0.0000**	12.97
46	cg26367439	10:44701964		*HNRNPA3P1*	**1.57E-04**	-5.91	0.1400	2.01	**0.0119**	3.89
47	cg16262898	1:3554782	*WDR8*	*WRAP73*	**1.59E-04**	-5.37	**0.0279**	2.53	0.0671	2.84
48	cg22971216	2:47572939		*CALM2*	**1.60E-04**	4.18	**0.0009**	-3.55	0.4348	-0.62
49	cg17158913	1:10764886	*CASZ1*	*CASZ1*	**1.65E-04**	-7.10	0.9424	-0.09	**0.0006**	7.20
50	cg15863827	10:79363895	*KCNMA1*	*KCNMA1*	**1.82E-04**	-5.39	0.3588	1.30	**0.0020**	4.10
51	cg05315047	3:137766727		*CLDN18*	**1.82E-04**	-5.82	**0.0048**	3.76	0.1394	2.06
52	cg09160955	15:89154092	*MIR7-2*	*DET1*	**1.91E-04**	-5.99	0.5126	1.22	**0.0275**	4.76
53	cg14040722	20:37229509	*C20orf95*	*ARHGAP40*	**1.92E-04**	-7.81	**0.0322**	2.78	**0.0056**	5.03
54	cg21554034	2:127785512		*BIN1*	**1.93E-04**	-6.01	0.0597	2.84	0.0522	3.17
55	cg23648516	13:78467552		*EDNRB*	**1.97E-04**	-9.49	0.6418	0.70	**0.0001**	8.79
56	cg14498666	9:21031822	*PTPLAD2*	*PTPLAD2*	**2.02E-04**	-3.64	0.0866	1.27	**0.0108**	2.37
57	cg11950360	18:34834690	*BRUNOL4*	*TPGS2*	**2.04E-04**	-7.70	**0.0450**	2.89	**0.0123**	4.81
58	cg01956614	15:62838506		*C2CD4B*	**2.05E-04**	4.19	0.8577	-0.19	**0.0004**	-4.01
59	cg22026192	12:124944377	*NCOR2*	*NCOR2*	**2.13E-04**	-7.81	**0.0197**	2.70	**0.0088**	5.11

Probe Information					5hmC		uC		5mC	
Rank	Probe	Position	UCSC Gene Annotation	GREAT Annotation	*P*	Δ	*P*	Δ	*P*	Δ
60	cg08570686	11:2287843		*TH*	**2.15E-04**	-5.34	**0.0302**	2.09	**0.0100**	3.26
61	cg14020824	4:106473040	*FLJ20184*	*ARHGEF38*	**2.17E-04**	-5.58	**0.0063**	4.09	0.3157	1.50
62	cg12997187	12:116382890		*MED13L*	**2.22E-04**	-7.09	0.1483	2.73	**0.0483**	4.36
63	cg14287311	13:77289721		*KCTD12*	**2.25E-04**	-8.36	**0.0393**	3.72	**0.0155**	4.64
64	cg04117455	14:104192737	*ZFYVE21*	*ZFYVE21*	**2.26E-04**	-7.58	**0.0008**	5.96	0.4946	1.62
65	cg02481256	19:11465868	*LPPR2*	*LPPR2*	**2.27E-04**	2.69	0.1779	-0.96	**0.0387**	-1.73
66	cg07016094	7:11676721	*THSD7A*	*THSD7A*	**2.29E-04**	-8.06	0.3330	1.63	**0.0006**	6.43
67	cg24372829	4:154702806	*SFRP2*	*RNF175*	**2.37E-04**	-5.29	**0.0260**	1.99	**0.0131**	3.31
68	cg07016258	15:83953929	*BNC1*	*BNC1*	**2.37E-04**	-3.47	0.1209	1.63	0.0739	1.84
69	cg14095316	4:100356538	*ADH7*	*ADH7*	**2.40E-04**	-5.12	0.2231	1.14	**0.0009**	3.98
70	cg13246744	13:91826938		*GPC5*	**2.49E-04**	-5.35	0.6268	-0.78	**0.0007**	6.13
71	cg24368226	15:100731456	*ADAMTS17*	*LYSMD4*	**2.49E-04**	-9.71	0.0775	2.85	**0.0029**	6.86
72	cg20706196	1:109242775	*PRPF38B*	*FNDC7*	**2.57E-04**	-7.77	**0.0049**	4.59	**0.0286**	3.18
73	cg17120570	6:736783		*FOXQ1*	**2.63E-04**	-9.95	0.0548	3.51	**0.0089**	6.44
74	cg24079381	17:77115473	*HRNBP3*	*ENGASE*	**2.64E-04**	-5.10	0.2994	1.48	**0.0425**	3.63
75	cg12835599	7:151526981	*PRKAG2*	*RHEB*	**2.67E-04**	-6.35	0.7299	0.37	**0.0009**	5.98
76	cg16018154	17:78863570	*RPTOR*	*CHMP6*	**2.78E-04**	5.36	0.1033	-1.71	**0.0061**	-3.65
77	cg05645927	6:27344393	*ZNF204P*	*ZNF204P*	**2.83E-04**	-7.57	0.7755	-0.48	**0.0000**	8.05
78	cg19312404	12:104213449	*NT5DC3*	*NT5DC3*	**2.83E-04**	5.05	**0.0365**	-2.26	**0.0093**	-2.79
79	cg00423598	7:156296491		*SHH*	**2.85E-04**	8.44	**0.0019**	-5.77	0.1509	-2.68
80	cg09045655	12:54392988	*HOXC9*	*HOXC9*	**2.88E-04**	-2.72	**0.0267**	2.19	0.6288	0.54
81	cg24110966	6:2968851	*SERPINB6*	*SERPINB9*	**2.94E-04**	-7.87	**0.0495**	3.14	**0.0169**	4.73
82	cg20702417	2:25538295	*DNMT3A*	*POMC*	**2.95E-04**	4.68	**0.0029**	-3.74	0.3581	-0.94
83	cg08368973	11:61519778	*C11orf9*	*C11orf9*	**3.01E-04**	-2.04	**0.0017**	2.26	0.6900	-0.22
84	cg05895618	11:19222395	*CSRP3;CSRP3*	*CSRP3*	**3.03E-04**	5.35	0.2942	-1.36	**0.0087**	-3.99
85	cg13186130	11:2321889	*C11orf21;TSPAN32*	*TSPAN32*	**3.04E-04**	-4.45	0.9878	0.03	**0.0360**	4.42
86	cg11493571	7:83638285	*SEMA3A*	*SEMA3E*	**3.21E-04**	-7.95	0.0577	3.68	**0.0227**	4.27
87	cg02958327	16:745663	*FBXL16*	*METRN*	**3.26E-04**	-7.47	0.6339	-0.40	**0.0004**	7.87
88	cg07932199	12:112008034	*ATXN2*	*ATXN2*	**3.26E-04**	-5.37	0.5867	0.65	**0.0026**	4.72
89	cg12411704	7:1786484	*ELFN1*	*ELFN1*	**3.28E-04**	-9.09	**0.0176**	3.97	**0.0382**	5.12
90	cg10157954	11:6951803	*ZNF215*	*ZNF215*	**3.32E-04**	-6.84	**0.0108**	4.00	0.0598	2.84
91	cg12284171	11:129814770	*PRDM10*	*NFRKB*	**3.32E-04**	-9.24	0.5825	0.94	**0.0011**	8.30

Probe Information					5hmC		uC		5mC	
Rank	Probe	Position	UCSC Gene Annotation	GREAT Annotation	*P*	Δ	*P*	Δ	*P*	Δ
92	cg20246315	6:30166614	*TRIM26*	*TRIM10*	**3.38E-04**	-6.90	0.4404	0.67	**0.0015**	6.23
93	cg13772070	9:108323097	*FKTN*	*TAL2*	**3.38E-04**	-7.23	**0.0466**	3.45	**0.0137**	3.78
94	cg21677258	6:30856693	*DDR1*	*DDR1*	**3.39E-04**	5.08	0.3441	-1.54	0.0530	-3.53
95	cg25347698	2:219381537	*USP37*	*USP37*	**3.46E-04**	-8.77	**0.0313**	4.25	**0.0195**	4.52
96	cg05697274	1:230415377	*GALNT2*	*PGBD5*	**3.53E-04**	-9.44	0.1085	2.10	**0.0038**	7.34
97	cg00668103	7:100084730	*C7orf51*	*AGFG2*	**3.56E-04**	-5.80	0.4227	1.04	**0.0050**	4.76
98	cg05988143	3:197488528	*FYTTD1*	*LRCH3*	**3.56E-04**	-5.82	0.1960	1.66	**0.0015**	4.17
99	cg26157031	11:102208366	*BIRC3*	*BIRC2*	**3.61E-04**	-6.37	0.2259	1.71	**0.0114**	4.66
100	cg26427535	1:240783389		*GREM2*	**3.68E-04**	-5.40	0.0999	2.59	0.0951	2.81

Supplementary Table 3: The 100 most significant neuropathology-associated differentially unmodified cytosine positions (DUPs) in the EC. Shown for each probe are chromosomal location (hg19), UCSC annotation, GREAT annotation, with corrected effect size (difference between Braak 0 and Braak VI (Δ) in unmodified cytosine levels (uC)) and corresponding P value after adjusting for the co-variates of age, sex and neuron/glia proportions. Shown for all probes are Δ and corresponding P value for 5mC and 5hmC levels. Probes where the 5hmC was below the level of detection are represented by "-". All P values <0.05 are shown in bold.

Probe information					Unmodified C		5mC		5hmC	
Rank	Probe	Position	UCSC Gene Annotation	GREAT Annotation	*P*	Δ	*P*	Δ	*P*	Δ
1	cg13617301	14:105619701	*JAG2*	*JAG2*	**4.94E-07**	4.19	**0.025**	-2.30	**0.0384**	-1.90
2	cg01091156	16:87991122	*BANP*	*BANP*	**1.66E-06**	3.96	**0.014**	-4.15	0.9017	0.19
3	cg21774827	19:54379120	*MYADM*	*PRKCG*	**2.35E-06**	-5.74	0.098	3.95	0.4183	1.80
4	cg26925343	16:28857904	*TUFM*	*SH2B1*	**3.22E-06**	1.44	**4.99E-04**	-1.15	-	-
5	cg17304222	5:180076905	*FLT4*	*FLT4*	**3.44E-06**	1.12	**2.85E-06**	-0.98	-	-
6	cg23898827	2:122096183	*CLASP1*	*TFCP2L1*	**1.14E-05**	-3.55	**7.01E-03**	4.99	0.4297	-1.44
7	cg06898199	7:128502890	*ATP6V1F*	*ATP6V1F*	**1.17E-05**	-1.03	**4.10E-05**	0.97	-	-
8	cg27642528	10:88814651	*GLUD1*	*GLUD1*	**1.20E-05**	-3.64	0.505	0.90	**0.0310**	2.74
9	cg02559264	11:73490474		*MRPL48*	**1.33E-05**	2.13	**6.51E-03**	-1.43	-	-
10	cg11563844	13:33780307	*STARD13*	*STARD13*	**1.89E-05**	-2.91	0.150	0.86	-	-
11	cg03309308	16:68525329		*SMPD3*	**2.23E-05**	4.33	**5.22E-03**	-3.14	0.2826	-1.19
12	cg05949331	8:143666563		*ARC*	**2.35E-05**	7.95	0.276	-3.00	0.0539	-4.95
13	cg04658038	17:64800166	*PRKCA*	*CACNG5*	**2.46E-05**	-4.36	0.573	1.11	0.1311	3.25
14	cg05376904	17:76920464	*TIMP2*	*TIMP2*	**2.48E-05**	-3.49	**0.022**	1.19	-	-
15	cg19965589	15:52043121	*TMOD2;LYSMD2*	*TMOD2*	**2.69E-05**	4.46	0.073	-2.68	0.2272	-1.78
16	cg03734026	22:50622440		*PANX2*	**2.78E-05**	-6.18	**5.08E-03**	4.92	0.4995	1.26
17	cg01042637	3:130743723	*ASTE1*	*ASTE1*	**3.13E-05**	-5.11	0.401	1.52	0.1012	3.59
18	cg17956832	17:45893828	*OSBPL7*	*OSBPL7*	**3.55E-05**	4.47	0.161	-1.47	**0.0116**	-2.99
19	cg04002944	8:143558609	*BAI1*	*BAI1*	**3.57E-05**	6.06	**4.44E-05**	-7.51	0.3478	1.46
20	cg27089736	2:3714278	*ALLC*	*ALLC*	**3.62E-05**	7.30	**1.94E-04**	-6.68	0.6228	-0.62
21	cg03975188	7:148131599		*CUL1*	**4.03E-05**	-7.21	0.564	1.11	**0.0124**	6.11
22	cg10614021	12:14134486	*GRIN2B*	*GRIN2B*	**4.07E-05**	-1.21	0.112	0.49	-	-
23	cg01348086	5:176785296	*RGS14*	*RGS14*	**4.14E-05**	-3.38	**1.23E-03**	2.82	0.5165	0.56
24	cg16013966	11:62380545	*EML3;ROM1*	*EML3*	**4.24E-05**	-3.47	0.838	-0.12	**5.96E-05**	3.59
25	cg13617837	6:3724690	*C6orf145*	*PXDC1*	**4.31E-05**	9.04	0.113	-4.70	0.0868	-4.34
26	cg27573549	19:56189952	*EPN1*	*EPN1*	**4.36E-05**	-4.61	0.291	2.80	0.5090	1.81
27	cg20326704	10:70321770	*TET1*	*TET1*	**4.47E-05**	-2.11	0.092	0.98	-	-

Probe information					Unmodified C		5mC		5hmC	
Rank	Probe	Position	UCSC Gene Annotation	GREAT Annotation	*P*	Δ	*P*	Δ	*P*	Δ
28	cg04346127	14:23590238		*CEBPE*	**4.74E-05**	-1.57	**6.94E-04**	1.39	-	-
29	cg27368828	10:72358359	*PRF1*	*PRF1*	**5.01E-05**	4.79	**8.70E-04**	-5.39	0.7100	0.60
30	cg17896776	4:172733760	*GALNTL6*	*GALNTL6*	**5.49E-05**	1.11	0.083	-0.40	-	-
31	cg21151057	7:157393764	*PTPRN2*	*DNAJB6*	**5.55E-05**	-5.22	0.777	0.52	0.0512	4.71
32	cg26954197	5:436816	*AHRR*	*C5orf55*	**5.79E-05**	4.57	0.068	-2.08	0.0533	-2.50
33	cg20618448	19:49962324	*ALDH16A1*	*ALDH16A1*	**6.01E-05**	-4.34	**1.25E-03**	6.31	0.1648	-1.96
34	cg02977761	19:42901279		*CNFN*	**6.49E-05**	0.99	**5.78E-05**	-1.12	-	-
35	cg20457962	11:3819153	*NUP98;PGAP2*	*NUP98*	**6.64E-05**	-1.16	**2.86E-04**	1.05	-	-
36	cg17554875	5:179520998		*RNF130*	**6.78E-05**	-6.19	0.677	0.67	**2.33E-03**	5.52
37	cg24903637	4:851793	*GAK*	*CPLX1*	**6.97E-05**	5.04	0.647	-0.73	**4.84E-03**	-4.31
38	cg04283864	6:4189186		*ECI2*	**7.32E-05**	-3.44	**3.52E-03**	3.56	-	-
39	cg03594868	19:18234829	*MAST3*	*IL12RB1*	**7.41E-05**	-6.74	0.153	4.44	0.3706	2.30
40	cg17867333	5:178423163	*GRM6*	*GRM6*	**7.48E-05**	5.31	**0.047**	-3.04	0.0554	-2.27
41	cg06679209	19:1073679	*HMHA1*	*HMHA1*	**7.54E-05**	-5.23	**0.020**	3.37	0.3164	1.86
42	cg16449840	12:54718606	*COPZ1*	*COPZ1*	**7.77E-05**	-1.27	**1.08E-04**	1.24	-	-
43	cg05044706	2:97526613	*SEMA4C*	*ANKRD39*	**8.54E-05**	5.47	0.211	-2.83	0.2140	-2.64
44	cg22842143	19:579592	*BSG*	*BSG*	**8.64E-05**	4.96	0.102	-4.07	0.6994	-0.89
45	cg09874873	6:160555573	*SLC22A1*	*SLC22A1*	**9.10E-05**	2.93	0.716	-0.38	**0.0196**	-2.55
46	cg03450049	22:50913555	*SBF1*	*SBF1*	**9.12E-05**	-0.52	**7.40E-03**	0.34	-	-
47	cg07674753	7:112758748	*LOC401397*	*ENSG00000214194*	**9.18E-05**	-1.98	**3.65E-04**	1.84	-	-
48	cg09391979	2:86333508	*PTCD3;POLR1A*	*PTCD3*	**9.31E-05**	-0.72	**2.12E-04**	0.63	-	-
49	cg16763574	13:111937052	*ARHGEF7*	*TEX29*	**9.33E-05**	5.81	**0.036**	-4.33	0.5457	-1.48
50	cg10092652	10:90967189	*CH25H*	*CH25H*	**9.33E-05**	0.57	**0.017**	-0.36	-	-
51	cg06653632	12:129281444	*SLC15A4*	*SLC15A4*	**9.60E-05**	-6.03	0.113	3.42	0.2189	2.61
52	cg18811158	19:649301	*RNF126*	*FGF22*	**1.01E-04**	5.76	**9.13E-03**	-5.68	0.9702	-0.08
53	cg05634040	19:1172418	*SBNO2*	*SBNO2*	**1.02E-04**	4.48	0.704	-0.55	**0.0128**	-3.93
54	cg27535215	10:49861457		*ARHGAP22*	**1.02E-04**	-3.96	0.063	3.22	0.7037	0.73
55	cg27375642	4:20960712	*KCNIP4*	*SLIT2*	**1.03E-04**	-4.24	0.990	0.02	**0.0205**	4.21
56	cg15322430	1:40157251	*HPCAL4*	*HPCAL4*	**1.03E-04**	-1.62	**6.95E-05**	1.71	-	-
57	cg23682913	1:2080710	*PRKCZ*	*C1orf86*	**1.04E-04**	4.50	**0.016**	-3.65	0.5508	-0.85
58	cg24723883	19:2608495	*GNG7*	*GNG7*	**1.08E-04**	5.69	0.330	-2.57	0.2337	-3.11
59	cg14911708	12:114404314	*RBM19*	*RBM19*	**1.08E-04**	-0.63	**1.70E-05**	0.68	-	-

Probe information					Unmodified C		5mC		5hmC	
Rank	Probe	Position	UCSC Gene Annotation	GREAT Annotation	*P*	Δ	*P*	Δ	*P*	Δ
60	cg03157738	19:11308118	*KANK2*	*KANK2*	**1.09E-04**	0.73	**4.56E-05**	-0.59	-	-
61	cg13851211	16:50321678	*ADCY7*	*ADCY7*	**1.12E-04**	-6.73	**8.39E-03**	5.36	0.5613	1.37
62	cg21999591	20:61898325		*ARFGAP1*	**1.14E-04**	-7.17	**9.29E-03**	5.99	0.6237	1.19
63	cg16389209	10:63809121	*ARID5B*	*ARID5B*	**1.14E-04**	3.01	**6.48E-04**	-1.86	-	-
64	cg15944459	22:38507857	*PLA2G6;BAIAP2L2*	*BAIAP2L2*	**1.15E-04**	4.96	**2.84E-03**	-3.62	0.3252	-1.34
65	cg08958618	13:102986478	*FGF14*	*FGF14*	**1.16E-04**	-3.74	0.794	-0.36	**0.0225**	4.10
66	cg23462505	10:1176149	*WDR37*	*IDI1*	**1.16E-04**	4.16	0.330	-1.72	0.2072	-2.44
67	cg05066959	8:41519308	*ANK1;MIR486*	*NKX6-3*	**1.18E-04**	-9.07	**1.13E-04**	13.10	0.1375	-4.04
68	cg14457311	17:48698799	*CACNA1G*	*ABCC3*	**1.19E-04**	-6.67	**0.048**	3.75	0.1616	2.92
69	cg05756220	11:30038685	*KCNA4*	*KCNA4*	**1.23E-04**	0.90	**0.036**	-0.47	-	-
70	cg12145550	20:44600942	*ZNF335*	*ZNF335*	**1.24E-04**	1.12	**1.28E-03**	-0.80	-	-
71	cg19893664	14:105619634	*JAG2*	*JAG2*	**1.26E-04**	6.33	0.184	-2.65	0.1035	-3.69
72	cg14088574	6:33234976	*VPS52*	*VPS52*	**1.27E-04**	3.28	0.608	-0.82	0.1357	-2.47
73	cg14218513	9:123639924	*PHF19*	*PHF19*	**1.29E-04**	-1.22	**8.29E-03**	0.81	-	-
74	cg00031340	16:81130212	*GCSH;LOC100329108*	*GCSH*	**1.30E-04**	-1.69	**1.19E-03**	1.43	-	-
75	cg21623722	19:14589148	*GIPC1*	*PTGER1*	**1.31E-04**	4.66	0.147	-3.40	0.5934	-1.26
76	cg00990022	1:40138052	*NT5C1A*	*NT5C1A*	**1.33E-04**	-2.72	0.204	0.61	-	-
77	cg08087102	12:108976666		*TMEM119*	**1.34E-04**	-5.39	0.071	4.10	0.5505	1.29
78	cg05977017	5:126856298	*PRRC1*	*PRRC1*	**1.38E-04**	3.87	0.184	-2.02	0.2472	-1.85
79	cg13713922	2:121625577	*GLI2*	*GLI2*	**1.39E-04**	1.99	**7.44E-04**	-1.55	-	-
80	cg02570361	17:42280026		*ATXN7L3*	**1.41E-04**	4.76	0.238	1.82	**1.35E-04**	-6.59
81	cg02627403	17:73823769	*UNC13D*	*UNC13D*	**1.44E-04**	-3.06	**0.031**	1.03	-	-
82	cg23418097	14:50155089	*POLE2*	*POLE2*	**1.46E-04**	1.03	**0.021**	-0.67	-	-
83	cg21721523	6:28545703	*SCAND3*	*SCAND3*	**1.47E-04**	3.49	0.443	1.03	**4.96E-03**	-4.51
84	cg24867180	15:42120426	*JMJD7-PLA2G4B;JMJD7*	*PLA2G4B*	**1.47E-04**	0.69	**0.033**	-0.41	-	-
85	cg15174949	19:6110595	*RFX2*	*RFX2*	**1.47E-04**	-0.41	**1.64E-03**	0.30	-	-
86	cg18981277	2:127805910	*BIN1*	*BIN1*	**1.48E-04**	4.24	0.169	-2.53	-	-
87	cg02814719	22:24200184	*SLC2A11*	*SLC2A11*	**1.49E-04**	0.87	**0.033**	-0.50	-	-
88	cg22720215	4:154048247		*TRIM2*	**1.56E-04**	4.62	0.210	2.28	**2.67E-03**	-6.90
89	cg19567193	15:65066231	*RBPMS2*	*RBPMS2*	**1.64E-04**	3.68	**0.020**	-4.71	0.6099	1.03
90	cg07226396	20:60813456	*OSBPL2*	*OSBPL2*	**1.64E-04**	-1.11	**1.13E-03**	0.87	-	-

Probe information					Unmodified C		5mC		5hmC	
Rank	Probe	Position	UCSC Gene Annotation	GREAT Annotation	*P*	Δ	*P*	Δ	*P*	Δ
91	cg04307702	2:3714315	*ALLC*	*ALLC*	**1.66E-04**	4.38	0.843	-0.26	**2.25E-03**	-4.12
92	ch.16.61261808F	16:62704307		*CDH8*	**1.68E-04**	-2.33	**0.002**	1.91	-	-
93	cg24698371	19:6110817	*RFX2*	*RFX2*	**1.68E-04**	-0.88	**1.36E-05**	0.97	-	-
94	cg01833057	4:7893062	*AFAP1*	*AFAP1*	**1.72E-04**	-5.41	0.736	-0.47	**1.07E-03**	5.88
95	cg15297628	11:69240844		*CCND1*	**1.75E-04**	-5.26	**5.44E-03**	5.41	0.9364	-0.14
96	cg19987840	4:123844099	*SPATA5;NUDT6*	*SPATA5*	**1.79E-04**	0.64	**0.050**	-0.31	-	-
97	cg10219575	19:59084223	*MZF1;LOC100131691*	*MZF1*	**1.80E-04**	-0.54	**9.21E-03**	0.35	-	-
98	cg09506504	6:106439790		*PRDM1*	**1.80E-04**	3.44	0.235	-1.10	**0.0343**	-2.33
99	cg14761246	3:182968758	*MCF2L2*	*B3GNT5*	**1.80E-04**	-5.47	**1.16E-03**	7.34	0.4000	-1.87
100	cg13745346	16:89044523	*CBFA2T3*	*CBFA2T3*	**1.81E-04**	-5.40	0.216	2.13	0.0772	3.27
