# Supplementary material for: Parallel profiling of DNA methylation and hydroxymethylation highlights neuropathology-associated epigenetic variation in Alzheimer’s disease
Source: Clin Epigenetics. 2019 Mar 21;11:52. doi: 10.1186/s13148-019-0636-y (PMC6429761; doi:10.1186/s13148-019-0636-y)

Figure S1 Quantile-quantile (Q-Q) plots of expected versus observed *P* value to check for inflation in linear regression analyses. Q-Q plots are shown for (**A**) unmodified cytosine (uC) (1-BS data), (**B**) 5mC (OxBS data), (**C**) 5hmC (BS – OxBS data) and (**D**) total DNA modifications (BS data).

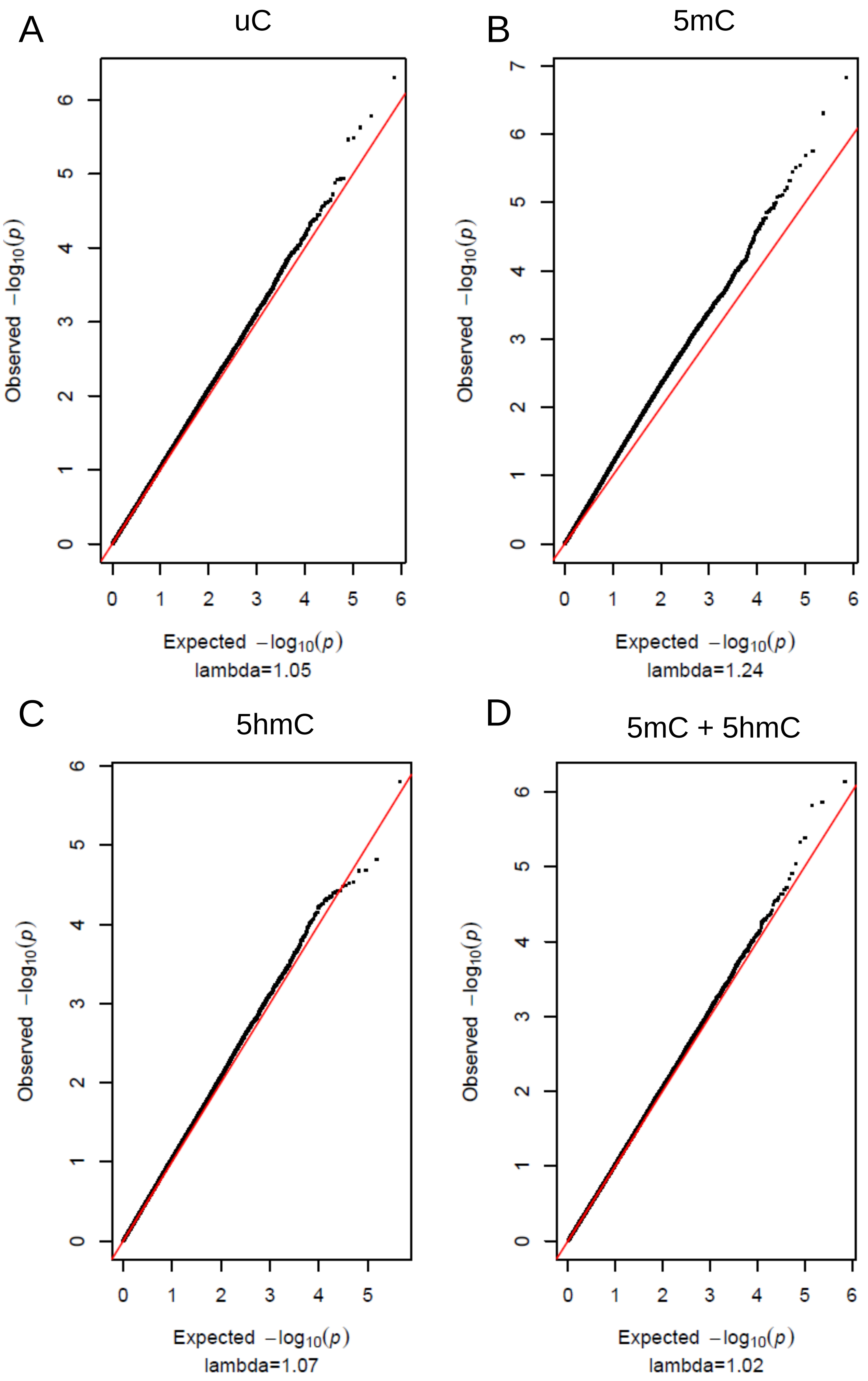

Supplement: Supplementary file 2 — Figure S1. Quantile-quantile (Q-Q) plots of expected versus observed P value to check for inflation in linear regression analyses. Q-Q plots are shown for (A) unmodified cytosine (uC) (1-BS data), (B) 5mC (OxBS data), (C) 5hmC (BS – OxBS data), and (D) total DNA modifications (BS data). (PDF 125 kb) [file 13148_2019_636_MOESM2_ESM.pdf]
